# A game-theoretic analysis of production and coordination under combined carbon policies

**DOI:** 10.1371/journal.pone.0336358

**Published:** 2026-04-29

**Authors:** Ao Qiao, Siyu Zeng, Jianing Wang

**Affiliations:** School of Logistics, Chengdu University of Information Technology, Chengdu, China; Harbin Institute of Technology, CHINA

## Abstract

To mitigate the growing threat of global warming, countries have increasingly adopted carbon policies to control industrial carbon emissions. Although previous research has analysed the effectiveness of individual carbon policies, such as cap-and-trade and subsidy schemes, the combined effects of these policies remain largely unexplored. Moreover, existing studies often overlook detailed production processes, such as lot-sizing decisions, which significantly influence total emissions of the manufacturer. To bridge these gaps, this study examines the coordination between a manufacturer and a retailer under a combined carbon policy framework, incorporating both cap-and-trade and low-carbon subsidy policies. A Stackelberg game model is developed to explore the strategic decisions of supply chain members. The manufacturer’s production process is modelled using queuing theory to capture the impact of operational details on carbon emissions. Supply chain scenarios are compared and analysed under different combinations of carbon policies and contractual arrangements. The results demonstrate that a combined carbon policy can enhance both profitability and sustainability across the supply chain. Among subsidy schemes, the green investment subsidy is more effective than product subsidy in emissions reduction. Additionally, while revenue-sharing contracts yield greater emissions reductions, cost-sharing contracts offer a better balance between economic and environmental objectives. These findings offer practical implications for policymakers and supply chain managers aiming to design integrated strategies that simultaneously achieve profitability and sustainability.

## 1. Introduction

A report by the World Meteorological Organization states that global emissions continued to increase in 2025, which highlights the need to intensify efforts to reduce carbon emissions [1]. Some countries have applied various carbon policies to reduce the carbon emissions of industrial activities, as the manufacturing industry is a major contributor to global carbon emissions. For instance, China has implemented the cap-and-trade policy that allows companies to purchase carbon credits if their emissions exceed regulatory limits or to sell surplus allowances to other companies. In addition, China has introduced subsidies to stimulate low-carbon investments, which have been shown to be more effective at reducing emissions than traditional carbon taxes [2,3]. While a significant body of literature has focused on the effects of individual carbon policies, only a limited number of studies have examined the interaction and combined impact of multiple policy instruments [4]. Understanding such combined effects is essential for designing more effective and comprehensive carbon abatement strategies. Moreover, the rapid growth of consumer green awareness has highlighted the potential profit of greener products, which also stimulates companies to engage in carbon emissions management [5].

Carbon emissions exist in every stage of a product’s life cycle, and the green efforts of manufacturers alone are often insufficient to achieve substantial emission reductions. As a result, sustainable coordination across the supply chains is crucial for reducing the total emissions. Some researchers have studied coordination in the green supply chain. For instance, Heydari et al. analysed the pricing issues of a green supply chain using green bonds [6]. Modak et al. developed three game-theoretical models to explore the decision-making of green supply chain parties [7]. Most previous studies focused on decision-making on green investment, pricing and order quantity issues. However, the impact of detailed production processes, particularly within the manufacturer’s operations, has received comparatively little attention. In practice, production planning parameters such as lot size have a significant influence on the carbon emissions of the manufacturer. Lot size determines the lead time for batch production, which in turn affects work-in-process (WIP) inventory levels and the associated carbon emissions. Optimising lot size not only helps minimise production costs but also enables better control of carbon emissions from WIP inventory. Integrating lot size optimisation with green investment decisions can therefore support emission reduction at the operational level and contribute to more fundamental carbon control. Despite its importance, the intersection of lot sizing and carbon emissions management remains underexplored. For instance, Wang and Choi studied lot sizing for a manufacturer with a make-to-order (MTO) production mode under the emission trading system [8]. Absi et al. explored lot sizing for a single product under a periodic carbon emissions constraint [9]. Nevertheless, the above studies only focused on the production strategy of a manufacturer and do not address coordination with other supply chain members. Although Qiao et al. studied supply chain coordination in lot sizing and green investment decision-making, they did not analyse the impact of carbon policies [10]. Therefore, it is important to further explore lot sizing decisions in a green supply chain under combined carbon policy scenarios.

To address these issues, this paper proposes a Stackelberg game model to study lot sizing, pricing and green investment issues in a green supply chain consisting of one manufacturer and one retailer under MTO production mode. It aims to explore the impact of the combined carbon policy (cap-and-trade and subsidy) on supply chain performance. In the proposed model, the manufacturer is the leader, which is responsible for the production planning for a single product produced in batches with the same lot size. Since the materials need to be gathered and then wait to be manufactured one by one, the waiting and operating processes in the production line are simulated using queuing theory. The retailer is the follower, which will decide the retail price considering the impact of consumer green awareness and price sensitivity on product demand. Two combined policy scenarios are proposed: the combination of cap-and-trade policy and green investment subsidy, and the combination of cap-and-trade policy and product subsidy. Moreover, green investment cost-sharing and revenue-sharing contracts are applied to the proposed model.

This study aims to answer the following research questions:

1. To what extent do detailed production parameters, specifically lot size and lead time, affect the carbon emission outcomes in a make-to-order (MTO) production environment?
2. How do the combinations of cap-and-trade regulation and low-carbon subsidies influence the strategic decisions and overall performance of a green supply chain?
3. Which contractual arrangement (cost-sharing or revenue-sharing) better aligns the economic interests of the manufacturer and retailer with environmental sustainability goals?

The originality and contributions of this paper are summarised as follows:

1. This study investigates the coordination and optimisation of a green supply chain under combined carbon policies. It contributes to the field by developing a novel two-layer model that links strategic supply chain coordination with detailed production process planning. Unlike previous works that treat lead time as fixed, this study applies queuing theory to model lead time and carbon emissions as functions of the production lot size, providing a more realistic framework for operational-level emission control.
2. The findings demonstrate that a combined carbon policy framework consistently enhances both profitability and environmental sustainability compared to individual policies. The green investment subsidy is found to be more effective at reducing emissions than the product subsidy. Furthermore, the analysis shows that consumer green awareness is an important factor that dictates the efficacy of carbon policies.
3. This study evaluates the role of contractual arrangements in incentivizing green efforts. It concludes while revenue-sharing contract generates greater emissions reductions, cost-sharing contracts provide a superior balance between carbon emission reduction and profitability protection of both supply chain parties.

The rest of this paper is organised as follows. Section 2 provides a review of relevant literature. In Section 3, the supply chain models under two combined carbon policy scenarios are formulated and analysed. Models for cost-sharing and revenue-sharing contracts are also presented in this section. A numerical case study is conducted in Section 4. The numerical results in various scenarios are compared and analysed in this section. Finally, in Section 5, conclusions and managerial insights are discussed and directions for future work are provided.

## 2. Literature review

The relevant research can be summarised into two main streams: supply chain coordination under a single carbon policy, and supply chain coordination under combined carbon policies.

### 2.1 Supply chain coordination under a single carbon policy

Some researchers have studied the influence of cap-and-trade policy in recent years [11]. For instance, Liu et al. compared the supply chain performance in three scenarios where different parties undertook emission reduction under cap-and-trade mechanism [12]. Some studies analysed coordination between the manufacturer and the retailer under different supply chain scenarios in the context of cap-and-trade policy [13,14]. For instance, Li et al. studied the impact of cap-and-trade policy on supply chain coordination under three types of contracts [15]. Yan et al. compared the supply chain performance without and with strategic inventory under a cap-and-trade scheme [16]. Some researchers have focused on the coordination between suppliers and manufacturers, such as the study of Kang and Tan [17].

Some other researchers focused on the impact of subsidies on green investment in the supply chain. For instance, Zhang and Yousaf analysed the petroleum industry in the context of government intervention and consumer green awareness [18]. He et al. investigated how government subsidy schemes and information asymmetry impact emission reduction and low-carbon promotion decisions in a green supply chain [19]. Moreover, some studies compared the impact of subsidies and other policies on supply chain profitability and sustainability [20]. Quayson et al. compared the impact of carbon subsidies and carbon taxes under price caps for the African energy industry by applying an optimisation algorithm [21].

Although previous studies have explored the green supply chain coordination in pricing, green investment and order quantity, most have overlooked the potential impact of lot-sizing decisions on the carbon emission of production processes. Optimising lot size enables manufacturers to control both production costs and carbon emissions, highlighting the significant potential of integrating lot sizing with carbon emission management. However, research on lot-sizing optimisation under carbon policies remains limited, primarily focusing on single-policy scenarios [10]. Although Mridha and Sarkar analysed the decision-making of a biofuel supply chain under controllable lead time and advertisement-dependent demand under three single carbon emissions regulations scenarios, the impact of combined carbon policies is overlooked [22]. Sebatjane investigated lot-sizing optimisation in a cold supply chain under cap-and-trade and cap-and-offset regulations [23]. However, this research did not encompass the production phase of the supply chain, leaving the effects of these carbon policies on production planning unexplored. Consequently, an investigation into the influence of combined carbon policies from a production planning perspective is warranted.

### 2.2 Supply chain coordination under combined carbon policies

Some countries, such as China, have applied more than one type of carbon policy, which highlights the importance of exploring the effectiveness of combined carbon policies. Zhao et al. discussed the effectiveness of carbon tax, cap-and-trade and combined carbon policies with consideration of household utility and government budget constraint [24]. Hussain et al. studied the pricing issue under cap-and-trade and subsidy policies [2]. Zhou and Li used the data of 271 Chinese cities to prove that the combination of new energy demonstration city and low-carbon city policies can significantly reduce carbon emissions [25]. Muthusamy et al. discussed the decision-making in a supply chain for scarce perishable goods under eight policy combination scenarios [26]. Although existing research confirms the effectiveness of combined carbon policies in reducing supply chain emissions, their operational implications for production planning remain largely unexplored.

Most studies on green supply chain management have overlooked the potential impact of the lot sizing issue on the carbon emissions of product processes, which indeed affects the carbon abatement outcomes of a supply chain. Moreover, the implications of combined carbon policies for production planning remain largely unexplored. To address these issues, this paper investigates the joint decisions of lot sizing, pricing and green investment issues under the combination of cap-and-trade and subsidy policies. It considers the integration of optimisation at both production process level and supply chain coordination level, and examines how key production parameters, such as lead time, affect supply chain profitability and sustainability outcomes.

## 3. Model analysis

This section illustrates the proposed supply chain model under two combined policy scenarios: the combination of cap-and-trade and green investment subsidy, and the combination of cap-and-trade and product subsidy. Supply chain models under revenue-sharing and cost-sharing contracts are also proposed in this section.

### 3.1 Model formulation

**Fig 1:**
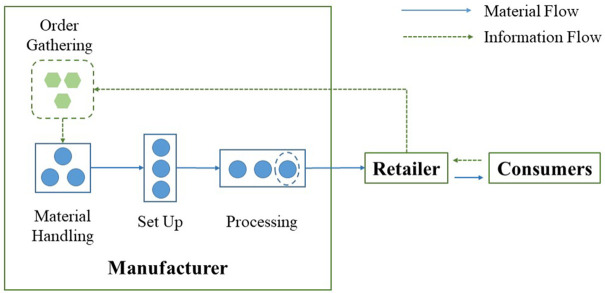
Supply chain model under MTO production.

Fig 1 illustrates the proposed supply chain model with one manufacturer and one retailer. The manufacturer is the leader in this Stackelberg game model, and the retailer is the follower. The manufacturer decides its lot size *Q*, and the emissions reduction amount *l* for each product, while the retailer decides the retail price *p*. Since this study focuses on the optimisation at the production line level, the transportation issue between these two parties is not considered to maintain a focused analysis on internal manufacturing efficiencies and their direct carbon footprint. In a green-sensitive market, product demand is influenced by retail price *p* and the product emissions reduction amount *l*. Achieving a higher emissions reduction amount can attract more green consumers, while a higher retail price may make some consumers tilt towards other substitutable products. Hence the product demand rate can be represented as *D* = *a* + *gl* − *bp*, where *a* is the original demand rate, *g* is the consumer green sensitivity, and *b* is the price sensitivity [18].

Production occurs under MTO production mode, so overproduction and order backlog are not considered. Production processes in the production line are simulated by queuing theory [27]. It simulates the processes in the production line where materials queue and wait to be manufactured one by one. All production line stages are mutually independent. When determining the expected lead time for individual parts, as was done in [28] and [29], machine breakdown is usually excluded from the calculation. Based on the study of Qiao, et al. [10], the total production lead time *E*(*T*) is defined as the sum of time spent in order gathering, material handling, setup and processing. The manufacturer starts production after the accumulated demand reaches batch size *Q*. The expected order gathering time *E*(*T*_*g*_) is (*Q* − 1)/2*D*. Raw materials and components are collected and sent to the production line for setup. The expected material handling time *E*(*T*_*B*_) is *B*(*Q* + 1)/2*.* Setup time for each batch is *τ*. Then, batches of materials will be manufactured one by one. The expected queuing time for processing *E*(*T*_*qp*_) is (*Q* − 1)/2*μ*. The expected processing time is 1/*μ*, where *μ* is the process rate. Therefore, the expected lead time is:


$$\begin{array}{c} E\left(T\right)=E\left(T_{g}\right)\text{+}E\left(T_{B}\right)+E\left(T_{S}\right)+E\left(T_{qp}\right)+E\left(T_{p}\right)\\ =\frac{Q-1}{2(a+gl-bp)}+\frac{B(Q+1)}{\text{2}}+\tau +\frac{Q+1}{2\mu } \end{array}$$ (1)

**Remark:** If the machine breakdown is considered, the proposed model can be modified as follows. Set the probability of machine breakdown to be *p*_*B*_, and the maintenance time to be *t*_*M*_. The expected lead time *E*(*T*) in Eq. (1) should be replaced by *E*(*T*)+ *p*_*B*_*t*_*M*_.

To ensure that the manufacturer’s production satisfies customer demand, the following constraint is necessary, which is:


$$\frac{Q}{E\left(T\right)}\geq a+gl-bp$$ (2)

It is formulated to ensure a minimum average production rate that aligns with market requirements. By centring the constraint on the mean expected value, the model can control the production rate in an acceptable range while ensuring computational tractability.

Under the cap-and-trade policy, a carbon cap *C* is set for the manufacturer for a certain period. The manufacturer must purchase carbon credits with a carbon price *P*_*E*_ for exceeding emissions. It can sell unused credits for profit on the carbon market. Two subsidy policies are involved in this study, which are the green investment subsidy and the green product subsidy. Under the green investment subsidy, the government provides a subsidy to cover part of the green investment cost for the manufacturer. The green investment cost of the manufacturer is *kl*^*2*^, where *k* is a cost parameter. The subsidy is *sl*^*2*^, where *s* is a subsidy ratio [30,31]. For the green product subsidy, the government provides a subsidy *m* to the manufacturer for each unit of product. The total emissions of the manufacturer *U* are the sum of emissions during production processes and the emissions of WIP inventories after green investment, which can be represented as:


$$U=e_{m}+e_{\text{1}}DH+e_{w}+e_{\text{2}}E\left(T\right)DH-l$$ (3)

where *e*_*m*_ and *e*_*w*_ are the fixed carbon emissions in production and WIP inventories, respectively. *e*_1_ is the variable production emissions per unit, and *e*_2_ is the variable carbon emissions for WIP inventory per unit per unit time.

### 3.2 Model under combined policy scenarios

This section studies the model analysis of supply chain models under two combined policy scenarios: the combination of cap-and-trade and green investment subsidy (Scenario 1), and the combination of cap-and-trade and product subsidy (Scenario 2).

#### 3.2.1 Scenario 1: C&T and green investment subsidy.

In this scenario, the government provides a subsidy to cover part of green investment cost of the manufacturer. The profit function of the retailer is:


$$\pi_{R}=(p-w-r)(a+gl-bp)H$$ (4)

The manufacturer’s profit function is:


$$\begin{array}{c} \pi_{M}=\left(w-\frac{S}{Q}-c-hE\left(T\right)\right)DH+P_{E}(C-U)-(k-s)l^{\text{2}}\\ =\left(w-c-P_{E}e_{\text{1}}-\frac{S}{Q}-(h+P_{E}e_{\text{2}})(\frac{B(Q+1)}{\text{2}}+\tau +\frac{Q+1}{2\mu })\right)(a+gl-bp)H\\ +P_{E}(C-e_{m}-e_{w}+l)-(k-s)l^{\text{2}}-\frac{(h+P_{E}e_{\text{2}})(Q-1)H}{\text{2}} \end{array}$$ (5)

Backward induction is applied to solve this Stackelberg game model. Taking the manufacturer’s decisions as given, the retailer sets the retail price to maximize its profit. Then by substituting the optimal retail price response into Eq. (5), the manufacturer’s optimal lot size and emissions reduction amount can be obtained.

**Proposition 1.1.** When 2*S*(*k-s*)(*a + gl-b*(*w + r*))*/Q*^*3*^ *> g*^*2*^*H*(*S/Q*^*2*^*-*(*h + P*_*E*_*e*_*2*_)(*B +* 1*/µ*)*/*2)^*2*^/4, the optimal lot size *Q*_g_* can be obtained by solving the following equation:


$$\begin{array}{c} \frac{g^{\text{2}}HQ^{\text{4}}(h+P_{E}e_{\text{2}}{)}^{\text{2}}}{16(k-s)}(B+\frac{\text{1}}{\mu }{)}^{\text{2}}-\left(\frac{Con}{\text{2}}(h+P_{E}e_{\text{2}})(B+\frac{\text{1}}{\mu })+h+P_{E}e_{\text{2}}\right)Q^{\text{3}}+SConQ-\frac{g^{\text{2}}HS^{\text{2}}}{4(k-s)}=0\\ s.t.\\ Q+1\geq (\frac{BQ}{\text{2}}+\frac{Q}{2\mu }+\frac{B}{\text{2}}+\tau +\frac{\text{1}}{2\mu })(a-b(w+r)+\frac{gP_{E}}{2(k-s)}\\ \text{+}\frac{g^{\text{2}}H}{4(k-s)}\left(w-c-P_{E}e_{\text{1}}-\frac{S}{Q}-(h+P_{E}e_{\text{2}})(\frac{B(Q+1)}{\text{2}}+\frac{Q+1}{2\mu }+\tau )\right)\\ where\;Con=a-b(w+r)+\frac{g^{\text{2}}H}{4(k-s)}\left(w-c-P_{E}e_{\text{1}}-(h+P_{E}e_{\text{2}})(\frac{B}{\text{2}}+\tau +\frac{\text{1}}{2\mu })\right)+\frac{gP_{E}}{2(k-s)} \end{array}$$ (6)

*Proof.* Proofs are given in the Appendix in S2 File.

Then the optimal emissions reduction amount *l*_g_* and retail price *p*_g_* can be obtained, which are:


$$\begin{array}{c} \overset{*}{\underset{g}{l}}=\frac{gH}{4(k-s)}\left(w-c-P_{E}e_{\text{1}}-\frac{S}{\overset{*}{\underset{g}{Q}}}-(h+P_{E}e_{\text{2}})(\frac{B\overset{*}{\underset{g}{Q}}+1}{\text{2}}+\frac{\overset{*}{\underset{g}{Q}}+1}{2\mu }+\tau )\right)+\frac{P_{E}}{2(k-s)}\\ \overset{*}{\underset{i}{p}}=\frac{a}{2b}+\frac{g\overset{*}{\underset{g}{l}}}{2b}+\frac{w+r}{\text{2}} \end{array}$$ (7)

#### 3.2.2 Scenario 2: C&T and green product subsidy.

In this scenario, the government provides a subsidy to the manufacturer for each unit of product. The profit function of the retailer is:


$$\pi_{R}=(p-w-r)(a+gl-bp)H$$ (8)

The profit function of the manufacturer is:


$$\begin{array}{c} \pi_{M}=\left(w-c+m-\frac{S}{Q}-hE\left(T\right)\right)DH+P_{E}(C-U)-kl^{\text{2}}\\ =\left(w-c+m-P_{E}e_{\text{1}}-\frac{S}{Q}-(h+P_{E}e_{\text{2}})(\frac{B(Q+1)}{\text{2}}+\tau +\frac{\left(Q+1\right)}{2\mu })\right)(a+gl-bp)H\\ +P_{E}(C-e_{m}-e_{w}+l)-\frac{(h+P_{E}e_{\text{2}})(Q-1)H}{\text{2}}-kl^{\text{2}}\\ s.t.\\ \frac{Q}{E\left(T\right)}\geq a+gl-bp \end{array}$$ (9)

Backward induction is also applied to solve the model in this scenario. Taking the manufacturer’s decisions as given, the retailer sets the retail price to maximize its profit based on Eq. (8). Then, by substituting the optimal retail price response into Eq. (9), the manufacturer’s optimal lot size and emissions reduction amount can be obtained.

**Proposition 1.2.** When 2*kS*(*a + gl* − *b*(*w + r*))*/Q*^*3*^ *> g*^*2*^*H*(*S/Q*^*2*^ − (*h + P*_*E*_*e*_*2*_)(*B +* 1*/µ*)*/*2)^*2*^*/*4, there exists an optimal lot size *Q*_p_*, which can be solved by the following equation:


$$\begin{array}{c} \frac{g(h+P_{E}e_{\text{2}}{)}^{\text{2}}}{8(k-P_{E})}(B+\frac{\text{1}}{\mu }{)}^{\text{2}}Q^{\text{4}}-(h+P_{E}e_{\text{2}})\left((\frac{B}{\text{2}}+\frac{\text{1}}{2\mu })Con+2\right)Q^{\text{3}}+SConQ-\frac{gS^{\text{2}}}{2(k-P_{E})}=0\\ s.t.\\ Q+1\geq (\frac{BQ+B}{\text{2}}+\frac{Q+1}{2\mu }+\tau )\left(\begin{array}{c} @l@\frac{a-b(w+r)}{\text{2}}\text{+}\frac{g}{2(k-P_{E})}(w-c-m-P_{E}e_{\text{1}}-\frac{S}{Q}\mathrm{\backslash vspace}1.5mm\\ -(h+P_{E}e_{\text{2}})(\frac{BQ+B}{\text{2}}+\frac{Q+1}{2\mu }+\tau ) \end{array}\right)\\ where\;Con=\frac{a-(w+r)b}{\text{2}}+\frac{g}{2(k-P_{E})}\left(w-c-P_{E}e_{\text{1}}+m-(h+P_{E}e_{\text{2}})(\frac{B}{\text{2}}+\tau +\frac{\text{1}}{2\mu })\right) \end{array}$$ (10)

*Proof.* Proofs are given in the Appendix in S2 File.

Then the optimal emissions reduction amount *l*_p_* and retail price *p*_p_* are:


$$\begin{array}{c} \overset{\text{*}}{\underset{p}{l}}=\frac{gH}{4k}\left(w-c-P_{E}e_{\text{1}}+m-\frac{S}{\overset{*}{\underset{p}{Q}}}-(h+P_{E}e_{\text{2}})(\frac{BQ_{p}\text{*}+B}{\text{2}}+\frac{Q_{p}\text{*}+1}{2\mu }+\tau )\right)+\frac{P_{E}}{2k}\\ \overset{*}{\underset{p}{p}}=\frac{a}{2b}+\frac{g\overset{\text{*}}{\underset{p}{l}}}{2b}+\frac{w+r}{\text{2}} \end{array}$$ (11)

### 3.3 Supply chain scenarios under contracts

To incentivize the manufacturer to make more efforts on carbon abatement, and ultimately achieve better performance of the whole supply chain, the cost-sharing and revenue-sharing contracts are applied between the manufacturer and the retailer. These two contracts can reduce the manufacturer’s financial pressure on green investment, so that it can achieve more emissions reduction and better profitability for the supply chain parties. These two types of contracts are widely applied in the renewable energy and automotive industries. For instance, wind turbine manufacturer Vestas works under the Power Purchase Agreements (PPAs), which include a revenue-sharing mechanism to share a portion of the revenue from the generated electricity [32]. Besides, Bayerische Motoren Werke (BMW) entered into a long-term contract with the Swedish battery developer Northvolt which involves a cost-sharing element [33]. Under the cost-sharing contract, the retailer takes *λ* proportion of the green investment cost to reduce the burden on the manufacturer. While the retailer shares *θ* proportion of its revenue with the manufacturer under the revenue-sharing contract. This section will analyse the impact of contracts on the supply chain scenarios discussed before. Table 1 summarises the notations for the optimal solution for decision variables (*Q*, *l* and *p*) under different scenarios.

**Table 1:** Decision variable notation.

	Cap-and-trade and green investment subsidy	Cap-and-trade and product subsidy
Cost-sharing	*Q*_*1*_, *l*_*1*_ and *p*_*1*_	*Q*_*3*_, *l*_*3*_ and *p*_*3*_
Revenue-sharing	*Q*_*2*_, *l*_*2*_ and *p*_*2*_	*Q*_*4*_, *l*_*4*_ and *p*_*4*_

#### 3.3.1 Contract application in Scenario 1.

1. Model with the cost-sharing contract

Since the retailer shares part of the manufacturer’s green investment cost, the green investment cost of the manufacturer turns to be (1 − *λ*)*kl*^*2*^. The retailer’s profit becomes:


$$\pi_{R}=(p-w-r)(a+gl-bp)H-\lambda kl^{\text{2}}$$ (12)

The profit function of the manufacturer is:


$$\begin{array}{c} \pi_{M}\text{=}\left(w-c-P_{E}e_{\text{1}}-\frac{S}{Q}-(h+P_{E}e_{\text{2}})(\frac{B(Q+1)}{\text{2}}+\tau +\frac{Q+1}{2\mu })\right)(a+gl-bp)H\\ -\left((1-\lambda )k-s\right)l^{\text{2}}+P_{E}(C-e_{m}-e_{w}+l)-\frac{(h+P_{E}e_{\text{2}})(Q-1)H}{\text{2}}\\ s.t.\\ \frac{Q}{E\left(T\right)}\geq a+gl-bp \end{array}$$ (13)

By applying the backward induction method, the manufacturer’s optimal lot size and emissions reduction amount can be obtained, as follows.

**Proposition 2.1.** When 2((1 − *λ*)*k-s*)*S*(*a + gl-b*(*w + r*))*/Q*^*3*^ *> g*^*2*^*H*(*S/Q*^*2*^ − (*h + P*_*E*_*e*_*2*_)(*B +* 1*/µ*)*/*2)^*2*^*/*4, the optimal lot size *Q*_*1*_ can be solved by the following equation:


$$\begin{array}{c} \frac{g^{\text{2}}H(h+P_{E}e_{\text{2}}{)}^{\text{2}}}{16(k-\lambda k-s)}(B+\frac{\text{1}}{\mu }{)}^{\text{2}}Q^{\text{4}}-(h+P_{E}e_{\text{2}})\left((\frac{B}{\text{2}}+\frac{\text{1}}{2\mu })Con+1\right)Q^{\text{3}}+SConQ-\frac{g^{\text{2}}S^{\text{2}}H}{4(k-\lambda k-s)}=0\\ s.t.\\ Q+1\geq (\frac{BQ}{\text{2}}+\frac{Q}{2\mu }+\frac{B}{\text{2}}+\tau +\frac{\text{1}}{2\mu })\left(\begin{array}{c} @l@a-b(w+r)+\frac{gP_{E}}{2(k-\lambda k-s)}\\ \text{+}\frac{g^{\text{2}}H}{4(k-\lambda k-s)}\left(w-c-P_{E}e_{\text{1}}-\frac{S}{Q}-(h+P_{E}e_{\text{2}})(\frac{B(Q+1)}{\text{2}}+\frac{Q+1}{2\mu }+\tau )\right) \end{array}\right)\\ where\;Con=a-b(w+r)+\frac{gP_{E}}{2(k-\lambda k-s)}\\ +\frac{g^{\text{2}}H}{4(k-\lambda k-s)}\left(w-c-P_{E}e_{\text{1}}-(h+P_{E}e_{\text{2}})(\frac{B}{\text{2}}+\tau +\frac{\text{1}}{2\mu })\right) \end{array}$$ (14)

*Proof.* Proofs are given in the Appendix in S2 File.

Then the optimal emissions reduction amount *l*_*1*_ and retail price *p*_*1*_ can be obtained, which are:


$$\begin{array}{c} l_{\text{1}}=\frac{gH}{4(k-\lambda k-s)}\cdot \left(w-P_{E}e_{\text{1}}-c-\frac{S}{Q_{\text{1}}}-(h+P_{E}e_{\text{2}})(\frac{BQ_{\text{1}}+B}{\text{2}}+\frac{Q_{\text{1}}+1}{2\mu }+\tau )\right)+\frac{P_{E}}{2(k-\lambda k-s)}\\ p_{\text{1}}=\frac{a}{2b}+\frac{gl_{\text{1}}}{2b}+\frac{w+r}{\text{2}}\\ \; \end{array}$$ (15)

1. Model with the revenue-sharing contract

The retailer gives *θ* proportion of its revenue to the manufacturer, so its profit becomes:


$$\pi_{R}=((1-\theta )p-w-r)(a+gl-bp)H$$ (16)

The manufacturer’s profit function is:


$$\begin{array}{c} \pi_{M}=\left(w-c-P_{E}e_{\text{1}}+\theta p-\frac{S}{Q}-(h+P_{E}e_{\text{2}})(\frac{B(Q+1)}{\text{2}}+\tau +\frac{Q+1}{2\mu })\right)(a+gl-bp)H\\ +P_{E}(C-e_{m}-e_{w}+l)-\frac{(h+P_{E}e_{\text{2}})(Q-1)H}{\text{2}}-(k-s)l^{\text{2}}\\ s.t.\\ \frac{Q}{E\left(T\right)}\geq a+gl-bp \end{array}$$ (17)

By applying the backward induction method, the manufacturer’s optimal lot size and emissions reduction amount can be obtained, as follows.

**Proposition 2.2.** When (2*k* − 2*s* − *θg*^*2*^*H/2b*)*S*(*a + gl* − *b*(*w + r*)/(1 − *θ*))*/Q*^*3*^ *> g*^*2*^*H*(*S/Q*^*2*^ − (*h + P*_*E*_*e*_*2*_)(*B + 1/µ*)*/*2)^*2*^*/*4 and *2*k − *2*s − *θg*^*2*^*H/*2*b* > 0, the optimal lot size *Q*_*2*_ can be obtained by the following equation:


$$\begin{array}{c} \frac{bg^{\text{2}}H(h+P_{E}e_{\text{2}}{)}^{\text{2}}}{16b(k-s)-4\theta g^{\text{2}}H}(B+\frac{\text{1}}{\mu }{)}^{\text{2}}Q^{\text{4}}-(h+P_{E}e_{\text{2}})\left((\frac{B}{\text{2}}+\frac{\text{1}}{2\mu })Con+1\right)Q^{\text{3}}+SConQ-\frac{bg^{\text{2}}HS^{\text{2}}}{4b(k-s)-\theta g^{\text{2}}H}=0\\ s.t.\\ Q+1\geq (\frac{BQ+B}{\text{2}}+\frac{Q+1}{2\mu }+\tau )\left(\begin{array}{c} @l@a-b(w+r)+\frac{2bgP_{E}}{4b(k-s)-\theta g^{\text{2}}H}\\ +\frac{\theta bg^{\text{2}}H}{8b^{\text{2}}(k-s)-2b\theta g^{\text{2}}H}(a-\frac{bw+br}{1-\theta })\text{+}\frac{bg^{\text{2}}H}{4b(k-s)-\theta g^{\text{2}}H}\left(Con2-\frac{S}{Q}-(h+P_{E}e_{\text{2}})(\frac{B(Q+1)}{\text{2}}+\frac{Q+1}{2\mu }+\tau )\right) \end{array}\right)\\ where\;Con=a-\frac{b(w+r)}{1-\theta }+\frac{bg^{\text{2}}H}{4b(k-s)-\theta g^{\text{2}}H}\left(Con2+\frac{\theta a}{2b}-\frac{\theta (w+r)}{2(1-\theta )}\right))\\ \;Con2=w-c-P_{E}e_{\text{1}}+\theta (\frac{a}{2b}+\frac{w+r}{2(1-\theta )})-(h+P_{E}e_{\text{2}})(\frac{B}{\text{2}}+\tau +\frac{\text{1}}{2\mu }) \end{array}$$ (18)

*Proof.* Proofs are given in the Appendix in S2 File.

Then the optimal emissions reduction amount *l*_*2*_ and retail price *p*_*2*_ can be achieved via:


$$\begin{array}{c} l_{\text{2}}=\frac{2bP_{E}}{4b(k-s)-\theta g^{\text{2}}H}+\frac{\theta gH}{8b(k-s)-2\theta g^{\text{2}}H}\left(a-\frac{b(w+r)}{1-\theta }\right)\\ \text{+}\frac{gbH}{4b(k-s)-\theta g^{\text{2}}H}\left(Con2-\frac{S}{Q_{\text{2}}}-(h+P_{E}e_{\text{2}})(\frac{B(Q_{\text{2}}+1)}{\text{2}}+\frac{Q_{\text{2}}+1}{2\mu }+\tau )\right)\\ p_{\text{2}}=\frac{a}{2b}+\frac{gl_{\text{2}}}{2b}+\frac{w+r}{2(1-\theta )}\\ where\;Con2=w-c-P_{E}e_{\text{1}}+\theta (\frac{a}{2b}+\frac{w+r}{2(1-\theta )})-(h+P_{E}e_{\text{2}})(\frac{B}{\text{2}}+\tau +\frac{\text{1}}{2\mu }) \end{array}$$ (19)

#### 3.3.2 Contract application in Scenario 2.

1. Model with the cost-sharing contract

In this scenario, the profit function of the retailer is:


$$\pi_{R}=(p-w-r)(a+gl-bp)H-\lambda kl^{\text{2}}$$ (20)

The profit function of the manufacturer is:


$$\begin{array}{c} \pi_{M}=\left(w-c+m-P_{E}e_{\text{1}}-\frac{S}{Q}-(h+P_{E}e_{\text{2}})(\frac{B(Q+1)}{\text{2}}+\tau +\frac{\left(Q+1\right)}{2\mu })\right)(a+gl-bp)H\\ +P_{E}(C-e_{m}-e_{w}+l)-\frac{(h+P_{E}e_{\text{2}})(Q-1)H}{\text{2}}-(1-\lambda )kl^{\text{2}}\\ s.t.\\ \frac{Q}{E\left(T\right)}\geq a+gl-bp \end{array}$$ (21)

**Proposition 2.3.** When 2*S*(1 − *λ*)*k*(*a + gl-b*(*w + r*))*/Q*^*3*^ *> g*^*2*^*H*(*S/Q*^*2*^ − (*h + P*_*E*_*e*_*2*_)(*B +* 1*/µ*)*/*2)^*2*^*/*4, the optimal lot size *Q*_*3*_ can be solved by the following equation:


$$\begin{array}{c} \frac{g^{\text{2}}H(h+P_{E}e_{\text{2}}{)}^{\text{2}}}{16(1-\lambda )k}(B+\frac{\text{1}}{\mu }{)}^{\text{2}}Q^{\text{4}}-(h+P_{E}e_{\text{2}})\left((\frac{B}{\text{2}}+\frac{\text{1}}{2\mu })Con+1\right)Q^{\text{3}}+SConQ-\frac{g^{\text{2}}S^{\text{2}}H}{4(1-\lambda )k}=0\\ s.t.\\ Q+1\geq (\frac{BQ+B}{\text{2}}+\frac{Q+1}{2\mu }+\tau )\left(\begin{array}{c} @l@a-b(w+r)+\frac{gP_{E}}{2(1-\lambda )k}\;\\ \text{+}\frac{g^{\text{2}}H}{4(1-\lambda )k}\left(w-c-m-P_{E}e_{\text{1}}-\frac{S}{Q}-(h+P_{E}e_{\text{2}})(\frac{BQ+B}{\text{2}}+\frac{Q+1}{2\mu }+\tau )\right) \end{array}\right)\\ where\;Con=a-(w+r)b+A\left(w-c-P_{E}e_{\text{1}}+m-(h+P_{E}e_{\text{2}})(\frac{B}{\text{2}}+\tau +\frac{\text{1}}{2\mu })\right)+\frac{gP_{E}}{2(1-\lambda )k} \end{array}$$ (22)

*Proof.* Proofs are given in the Appendix in S2 File.

Then the optimal emissions reduction amount *l*_*3*_ and retail price *p*_*3*_ can be calculated via the following equations:


$$\begin{array}{c} l_{\text{3}}=\frac{gH}{4(1-\lambda )k}\left(w-c-P_{E}e_{\text{1}}+m-\frac{S}{Q_{\text{3}}}-(h+P_{E}e_{\text{2}})(\frac{BQ_{\text{3}}+B}{\text{2}}+\frac{Q_{\text{3}}+1}{2\mu }+\tau )\right)+\frac{P_{E}}{2(1-\lambda )k}\\ p_{\text{3}}=\frac{a}{2b}+\frac{g}{2b}l_{\text{3}}+\frac{w+r}{\text{2}} \end{array}$$ (23)

1. Model with the revenue-sharing contract

In this case, the profit function of the retailer should be:


$$\pi_{R}=\left((1-\theta )p-w-r\right)(a+gl-bp)H$$ (24)

The profit function of the manufacturer is:


$$\begin{array}{c} \pi_{M}=\left(w-c+m+\theta p-P_{E}e_{\text{1}}-\frac{S}{Q}-(h+P_{E}e_{\text{2}})(\frac{B(Q+1)}{\text{2}}+\tau +\frac{\left(Q+1\right)}{2\mu })\right)(a+gl-bp)H\\ +P_{E}(C-e_{m}-e_{w}-l)-\frac{(h+P_{E}e_{\text{2}})(Q-1)H}{\text{2}}-kl^{\text{2}}\\ s.t.\\ \frac{Q}{E\left(T\right)}\geq a+gl-bp \end{array}$$ (25)

**Proposition 2.4.** When *S*(2*k-*θ*g*^*2*^*H/*2*b*)(*a + gl-b*(*w + r*)*/*(*1-θ*))*/Q*^*3*^ *> g*^*2*^*H*(*S/Q*^*2*^*-*(*h + P*_*E*_*e*_*2*_)(*B + 1/µ*)*/*2)^*2*^/4 and *k-θg*^*2*^*H/b >* 0*,* the optimal lot size *Q*_*4*_ can be solved via:


$$\begin{array}{c} \frac{A(h+P_{E}e_{\text{2}}{)}^{\text{2}}}{\text{4}}(B+\frac{\text{1}}{\mu }{)}^{\text{2}}Q^{\text{4}}-(h+P_{E}e_{\text{2}})\left((\frac{B}{\text{2}}+\frac{\text{1}}{2\mu })Con+1\right)Q^{\text{3}}+SConQ-AS^{\text{2}}=0\\ where\;A=\frac{bg^{\text{2}}H}{4bk-\theta g^{\text{2}}H}\\ Con=a-\frac{(w+r)b}{1-\theta }+\frac{2AP_{E}}{gH}+A\left(w-c+m-P_{E}e_{\text{1}}+\frac{\theta a}{b}-(h+P_{E}e_{\text{2}})(\frac{B}{\text{2}}+\tau +\frac{\text{1}}{2\mu })\right)\;\\ s.t.\\ Q+1\geq (\frac{BQ}{\text{2}}+\frac{Q}{2\mu }+\frac{B}{\text{2}}+\tau +\frac{\text{1}}{2\mu })\left(a-b(w+r)+\frac{2A}{gH}(P_{E}+\frac{g\theta H}{4b}(a-\frac{b(w+r)}{1-\theta })\right)\\ +A\left(w-c+m-P_{E}e_{\text{1}}+\frac{\theta a}{2b}+\frac{\theta (w+r)}{2(1-\theta )}-\frac{S}{Q}-(h+P_{E}e_{\text{2}})(\frac{BQ+B}{\text{2}}+\frac{Q+1}{2\mu }+\tau )\right) \end{array}$$ (26)

*Proof.* Proofs are given in the Appendix in S2 File.

Then the optimal emissions reduction amount *l*_*4*_ and retail price *p*_*4*_ can be achieved, which are:


$$\begin{array}{c} l_{\text{4}}=\frac{bgH}{4bk-\theta g^{\text{2}}}\left(w-c+m-P_{E}e_{\text{1}}+\frac{\theta a}{2b}+\frac{\theta (w+r)}{2(1-\theta )}-\frac{S}{Q_{\text{4}}}-(h+P_{E}e_{\text{2}})(\frac{BQ_{\text{4}}+B}{\text{2}}+\frac{Q_{\text{4}}+1}{2\mu }+\tau )\right)\\ +\frac{2b}{4bk-\theta g^{\text{2}}}\left(P_{E}+\frac{g\theta H}{4b}(a-\frac{b(w+r)}{1-\theta })\right)\\ p_{\text{4}}=\frac{a}{2b}+\frac{gl_{\text{4}}}{2b}+\frac{w+r}{2(1-\theta )} \end{array}$$ (27)

## 4. Numerical case studies

Numerical case studies are conducted on the supply chain models under different scenarios to analyse the impact of combined policy and several important parameters. Data collected from a machine tool company in China are applied in this paper [10].

### 4.1 Parameter settings and units

Parameter values applied in the proposed model are summarized in Table 2. The monetary unit is Renminbi (RMB), and the time unit is hours. The planning horizon is set to one year, calculated as 240 working days with 8 operating hours per day, which is H = 1920 hours in total. To ensure dimensional consistency in the carbon abatement calculations, all emission-related parameters originally measured in kilograms have been converted to tons [34].

**Table 2:** Parameter values and units.

Parameters	Description	Value	Unit
*Market and Demand Parameters*			
*a*	Demand rate	2	units/hour
*g*	Consumer green awareness	0.01	–
*b*	Price sensitivity	0.002	–
*w*	Product wholesale price	350	RMB/unit
*r*	Retailer operation cost	20	RMB/unit
*Production and Operations Parameters*			
*B*	Material handling time	0.05	hour/unit
*τ*	Setup time	0.05	hour/batch
*µ*	Process rate	15	units/hour
*S*	Setup cost	1000	RMB/batch
*h*	WIP holding cost	5	RMB/unit
*c*	Unit production cost	50	RMB/unit
*H*	Planning horizon	1920	hours
*Carbon Policy Parameters*			
*k*	Green investment cost coefficient	60	RMB/ton^2^
*s*	Green investment subsidy cost coefficient	10	RMB/ton^2^
*m*	Product subsidy	20	RMB/unit
*e*_*m*_	Fixed emission (production)	10	Ton/year
*e*_*w*_	Fixed emission (WIP inventory)	8	Ton/year
*e*_*1*_	Variable emission (production)	0.01	tons/unit
*e*_*2*_	Variable emission (WIP inventory)	0.0005	tons/unit/year
*P*_*E*_	Carbon price	23	RMB/ton
*C*	Carbon cap	30	tons

### 4.2 Analysis of results

#### 4.2.1 Supply chain under different carbon policy scenarios.

Table 3 presents the numerical results for four supply chain scenarios. Scenarios 1 and 2 serve as benchmark models of a supply chain without government subsidy. In Case 1, the manufacturer neglects green investment and relies exclusively on purchasing carbon credits from the market. In Case 2, the manufacturer conducts green investment while participating in carbon credit trading. The results for Scenarios 3 and 4 provide empirical validation for the theoretical frameworks established in Proposition 1.1 and Proposition 1.2, respectively.

**Table 3:** Supply chain performance under four cases.

Case	Lot size *Q*	Retail price *p*	Profit (10^5^RMB)		Subsidy (10^3^RMB)	Emission reduction (ton)	Emission in production (ton)	Emission in WIP inventory (ton)	Total Emission (ton)
			M	R					
1	15.43	685	2.10	3.81	0	0	22.10	15.39	37.49
2	16.50	732.59	2.32	5.05	0	19.04	23.92	16.00	20.92
3	16.70	742.28	2.36	5.32	5.23	22.91	24.29	16.12	17.55
4	16.58	736.65	2.60	5.16	28.15	20.66	24.08	16.05	19.50

It can be observed that an absence of green investment (Case 1) results in high total emission quantities, which require extra carbon credits. The adoption of green investment (Case 2) enhances profitability while simultaneously reducing total emissions. The introduction of governmental subsidies (Scenarios 3 and 4) further improves the profit and carbon abatement outcomes. Under the specified subsidy coefficient setting, the green investment subsidy (Scenario 3) leads to a larger lot size, a higher retail price and greater substantial emissions reductions compared to the product subsidy (Scenario 4). Although production and WIP inventory emissions are marginally higher under the investment subsidy, the investment subsidy helps the manufacturer achieve more emissions reduction, so the total emissions is smaller under the investment subsidy. The product subsidy facilitates higher manufacturer profit, while the retailer has higher profit under the investment subsidy. The investment subsidy helps the manufacturer achieve more emissions reductions, so the manufacturer can sell more credits. Critically, the investment subsidy achieves these superior environmental results with lower annual government expenditure. Therefore, the green investment subsidy is the optimal policy choice for governments seeking to maximize carbon abatement while maintaining fiscal responsibility.

**Fig 2:**
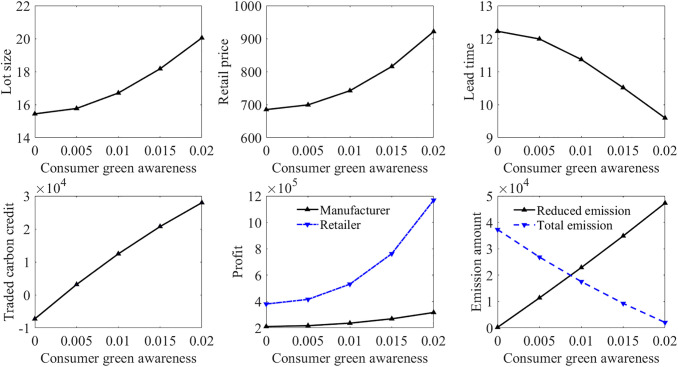
Impact of consumer green awareness in Case 3.

**Fig 3:**
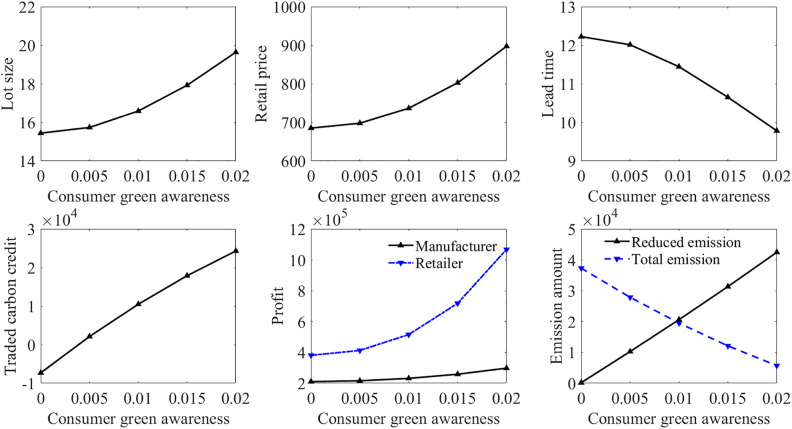
Impact of consumer green awareness in Case 4.

Consumer green awareness is an important factor that dictates the efficacy of carbon policies. Figs 2 and 3 show the influence of consumer green awareness on the models in Case 3 and Case 4, respectively. It can be observed that the increase in consumer green awareness consistently enhances supply chain performance across both carbon policy scenarios. Higher consumer green awareness permits the manufacturer to determine a larger lot size and a higher emission reduction level, and allows the retailer to set a higher retail price. Although a larger lot size makes the production time longer, the increased demand rate due to higher consumer green awareness reduces the order gathering time, so that the lead time becomes shorter. This green-sensitive demand effectively offsets the costs associated with green investment, suggesting that the success of carbon policies is heavily contingent upon the environmental consciousness of the consumer. By comparing Figs 2 and 3, it is found that increased consumer green awareness can improve the manufacturer’s profit more significantly under the green investment subsidy policy, and can also help the manufacturer achieve more emissions reductions. For policy design to reach peak efficiency, regulatory instruments should be coordinated with market-based incentives that bolster consumer green awareness. For instance, policy design should include non-monetary interventions, such as standardized green labelling and public education campaigns, to elevate consumer green awareness. By fostering a greener marketing environment, policymakers can reduce the industry’s reliance on direct subsidies, allowing market-based incentives to drive the transition toward sustainability.

**Fig 4:**
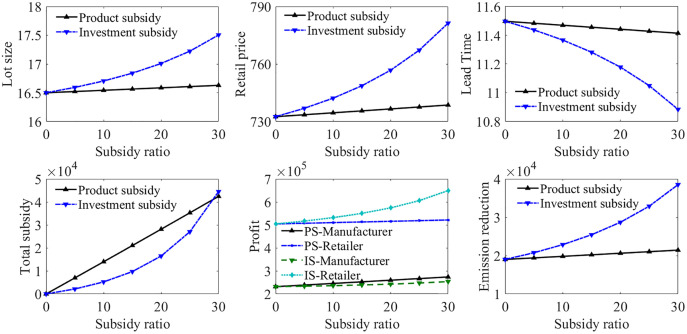
Impact of the subsidy ratio of investment and product subsidies.

Fig 4 shows the analysis of the impact of subsidy ratios. When the subsidy ratio is 0, it means no subsidy is applied. It can be observed that the application of both subsidy policies can motivate the manufacturer to achieve higher emission reduction, and improve the profit of both parties. The application of the subsidy helps the manufacturer achieve more emission reduction, which attracts more green consumers. The increment in demand caused by the manufacturer also makes the retailer decide on a higher retail price. Under the 1 year period, the investment subsidy can achieve more emission reduction than the product subsidy with less subsidy expenditure. Besides, increasing the investment subsidy ratio is an effective method to stimulate the manufacturer to achieve more carbon abatement. However, a higher subsidy ratio means the government has to spend more expenditure. The government should make a trade-off between the carbon abatement outcome and the financial expenditure.

#### 4.2.2 Supply chain models with contracts.

Numerical case studies are also conducted for the supply chain models in Case 3 and Case 4 under the cost-sharing and revenue-sharing contracts. In practice, since the application of contracts is to improve the supply chain performance, companies will set the contract ratio by analysing historical operational data and market forecasts to identify a win-win zone that both parties can accept. A sensitivity analysis across a broad range of values for contract parameters has been conducted to find a feasible region for coordination.

1. Contract application for the supply chain model in Case 3

**Fig 5:**
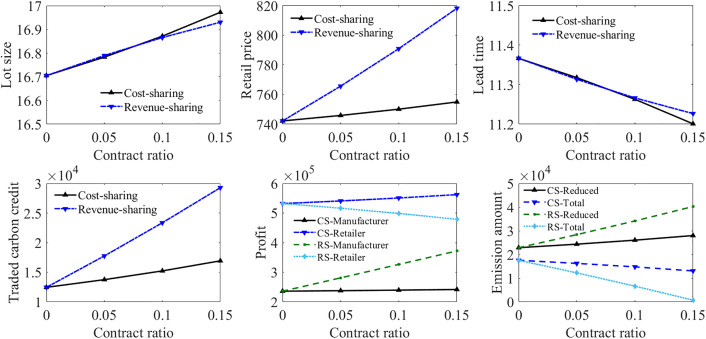
Contract application for supply chain model in Case 3.

The cost-sharing and revenue-sharing contracts are applied to the supply chain to help the manufacturer reduce its financial pressure in green investment, as shown in Fig 5. CS refers to the situation under the cost-sharing contract and RS refers to the situation under the revenue-sharing contract. CS-Reduced represents the emission reduction of the manufacturer under the cost-sharing contract, and CS-Total represents the total carbon emissions after green investment under the cost-sharing contract. Similarly, RS-Reduced represents the emission reduction of the manufacturer under the revenue-sharing contract, and RS-Total represents the total carbon emissions after green investment under the revenue-sharing contract.

When the contract ratio is 0, it means the decentralised supply chain scenario without any contract. It can be observed that the application of both contracts can alleviate the financial pressure of the manufacturer due to green investment, and help it achieve a higher emissions reduction amount. Under both contracts, a higher contract ratio makes the manufacturer determine a larger lot size, and enables the retailer to decide a higher retail price. The lead time also decreases due to the increased demand rate caused by the increased emissions reduction amount. Both contracts can increase the total emissions reduction amount of the manufacturer, while the total emissions of the manufacturer are reduced more obviously under the revenue-sharing contract than the cost-sharing contract with the increment of contract ratio. However, the revenue-sharing contract helps the manufacturer achieve higher profit, but makes the retailer suffer a profit loss. When the revenue-sharing contract ratio reaches 0.15, the manufacturer reaches the maximum emissions reductions amount. Hence further increasing the contract ratio will not improve the carbon abatement outcome. The emissions reduction amount of the manufacturer under the cost-sharing contract is smaller than that under the same revenue-sharing contract ratio. However, it can help both supply chain parties achieve more profit, though the profit increment of the manufacturer is not obvious.

1. Contract application for the supply chain model in Case 4

**Fig 6:**
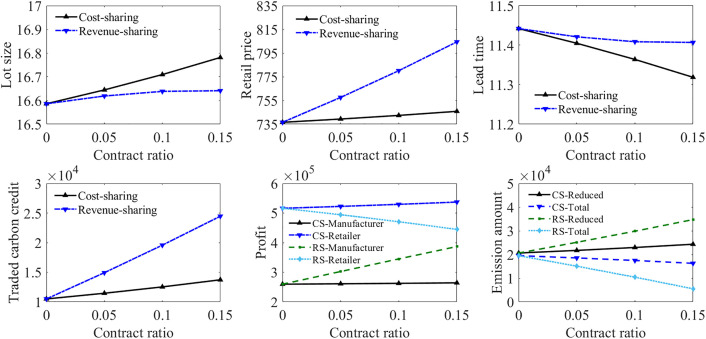
Contract application for supply chain model in Case 4.

Both contracts are applied to the supply chain model in Case 4, as shown in Fig 6. These two contracts have similar influence on the profitability and emissions abatement under the two combined carbon policy scenarios. The revenue-sharing contract leads to more emissions reduction and higher profit for the manufacturer, but makes the retailer suffer profit loss. The cost-sharing contract can help both parties achieve better profits, but is less effective in improving the emission reduction outcome compared with the revenue-sharing contract.

#### 4.2.3 Supply chain considering machine breakdown.

Table 4 shows the supply chain performance under consideration of machine breakdown, with the maintenance time to be 0.5 hours. It can be observed that there is a marginal increase in WIP inventory emissions and total emissions as probability rises, suggesting that higher uncertainty slightly offsets the outcomes from emissions abatement. As the probability increases from 0 to 0.05, the retail price shows a slight downward trend in both scenarios. Under both policy scenarios, the profitability for the manufacturer and the retailer remains relatively stable. Furthermore, increased uncertainty regarding machine breakdowns leads to a marginal decline in emission reduction levels, which subsequently results in a lower subsidy amount under the green investment policy.

**Table 4:** Supply chain performance considering machine breakdown.

Probability	Lot size *Q*	Retail price *p*	Profit (10^5^RMB)		Subsidy (10^3^RMB)	Emission reduction (ton)	Emission in production (ton)	Emission in WIP inventory (ton)	Total Emission (ton)
			M	R					
Under C&T and green investment subsidy									
0	16.70	742.28	2.36	5.32	5.23	22.91	24.29	16.12	17.55
0.01	16.70	742.16	2.36	5.32	5.23	22.86	24.29	16.13	17.55
0.05	16.70	742.14	2.36	5.32	5.22	22.86	24.29	16.14	17.57
Under C&T and product subsidy									
0	16.58	736.65	2.60	5.16	28.15	20.66	24.08	16.05	19.50
0.01	16.59	736.56	2.60	5.16	28.15	20.62	24.08	16.06	19.51
0.05	16.59	736.54	2.60	5.16	28.15	20.61	24.08	16.07	19.53

## 5. Conclusion and policy implications

This paper studies the coordination between a manufacturer and a retailer in lot sizing, green investment and pricing issues by applying queuing theory for the production processes of the manufacturer and the Stackelberg game model for the whole supply chain. It analyses the impact of combined carbon policy and consumer green awareness on the supply chain profitability and emissions reduction outcome, and discusses the effectiveness of the cost-sharing and revenue-sharing contracts in improving the supply chain performance. The combined carbon policy consists of the cap-and-trade policy and subsidy policy. Two types of low-carbon subsidies are studied in this paper, which are the green investment subsidy and product subsidy. It is proved that both subsidy policies can improve the supply chain performance in both sustainability and profitability. Besides, the cost-sharing contract can improve the emission reductions and the profitability of both supply chain parties simultaneously under both subsidy scenarios.

This study offers managerial insights for green supply chain participants and provides policy recommendations for government authorities, as detailed below.

1. Analytical results indicate that green investment subsidies outperform product subsidies by achieving greater emissions reductions with lower government expenditure. In the context of regional carbon markets, policymakers should link the subsidy to verified carbon abatement outcomes, which requires the support from the carbon accounting system. This alignment ensures fiscal efficiency by rewarding actual emission reductions. Furthermore, increasing subsidy levels is recommended, as higher financial incentives correlate directly with increased green efforts toward carbon abatement.
2. For managerial practice, particularly regarding small and medium-sized enterprises facing capital constraints, contract selection is critical. Although revenue-sharing contracts significantly enhance emission reduction, they yield suboptimal retailer profits. Therefore, the cost-sharing contract is a more viable supply chain mechanism, as it optimises both total profit and emission abatement. This collaborative framework allows manufacturers to mitigate the financial risks of green investment.
3. The efficacy of low-carbon policies relies heavily on a green-sensitive marketing environment. Both governmental and corporate stakeholders should implement non-monetary interventions, such as standardized green labelling and public education campaigns, to elevate consumer green awareness. Increased consumer green awareness allows manufacturers to capture a larger market share through emissions reduction, providing retailers the leverage to implement a higher retail price for green products without a corresponding reduction in demand.

The proposed model can be further developed in several directions. First, the proposed optimization model operates under the assumption of stable machine performance, excluding machine breakdowns. Future research could incorporate stochastic failure variables and maintenance scheduling to enhance the model’s robustness against real-time disruptions. Second, this study focuses on production-line-level carbon emissions, omitting the environmental impact of upstream and downstream logistics. The supply chain model can be expanded to a multi-echelon supply chain context for further investigation. Besides, possible green efforts of the retailer can also be considered in the study, such as using advertising or production promotion for green products. The variation in product demand can also be considered in the model in future research.

## Supporting information

S1 File Data set of numerical case study. (XLSX)

S2 File Appendix. (DOCX)

S1 Fig Graphical abstract. (TIFF)

## Abbreviations

BMW: Bayerische Motoren Werke
C&T: Cap-and-trade
CS: Cost-sharing
MTO: Make-to-order
PPAs: Power Purchase Agreements
RS: Revenue-Sharing
RMB: Renminbi (Chinese Currency)
WIP: Work-in-process

## References

[1] World Meteorological Organization. (2025). State of the Climate Update for COP30. Available from: https://library.wmo.int/records/item/69674-state-of-the-climate-update-for-cop30

[2] Hussain J, Pan Y, Ali G, Xiaofang Y. Pricing behavior of monopoly market with the implementation of green technology decision under emission reduction subsidy policy. Sci Total Environ. 2020;709:136110. doi: 10.1016/j.scitotenv.2019.136110 31905579

[3] Li F, Guo Y, Liu B. Impact of government subsidies and carbon inclusion mechanism on carbon emission reduction and consumption willingness in low-carbon supply chain. J Clean Prod. 2024;449:141783. doi: 10.1016/j.jclepro.2024.141783

[4] Zhang L, Ren J, Zhang G. Optimal dynamic strategy for emission reduction and operation considering hybrid carbon policy with carbon tax and cap-and-trade. Comput Indust Eng. 2024;187:109820. doi: 10.1016/j.cie.2023.109820

[5] García-Salirrosas EE, Escobar-Farfán M, Gómez-Bayona L, Moreno-López G, Valencia-Arias A, Gallardo-Canales R. Influence of environmental awareness on the willingness to pay for green products: an analysis under the application of the theory of planned behavior in the Peruvian market. Front Psychol. 2024;14:1282383. doi: 10.3389/fpsyg.2023.1282383 38282852 PMC10811795

[6] Heydari H, Taleizadeh AA, Jolai F. Financing a two-stage sustainable supply chain using green bonds: preventing environmental pollution and waste generation. Eng Appl Artif Intell. 2023;117:105583. doi: 10.1016/j.engappai.2022.105583

[7] Modak NM, Sinha S, Senapati T, Simic V, Pamucar D. Game theoretical analysis in two-echelon sustainable supply chains to manage and coordinate strategic decisions. Comput Indust Eng. 2024;192:110204. doi: 10.1016/j.cie.2024.110204

[8] Wang XJ, Choi SH. Stochastic lot sizing manufacturing under the ETS system for maximisation of shareholder wealth. Eur J Oper Res. 2015;246(1):66–75. doi: 10.1016/j.ejor.2015.04.021

[9] Absi N, Dauzère-Pérès S, Kedad-Sidhoum S, Penz B, Rapine C. The single-item green lot-sizing problem with fixed carbon emissions. Eur J Oper Res. 2016;248(3):849–55. doi: 10.1016/j.ejor.2015.07.052

[10] Qiao A, Choi SH, Wang XJ. Lot size optimisation in two-stage manufacturer-supplier production under carbon management constraints. J Clean Prod. 2019;224:523–35. doi: 10.1016/j.jclepro.2019.03.232

[11] Xu S, Govindan K, Wang W, Yang W. Supply chain management under cap-and-trade regulation: a literature review and research opportunities. Int J Prod Econ. 2024;271:109199. doi: 10.1016/j.ijpe.2024.109199

[12] Liu H, Kou X, Xu G, Qiu X, Liu H. Which emission reduction mode is the best under the carbon cap-and-trade mechanism?. J Clean Prod. 2021;314:128053. doi: 10.1016/j.jclepro.2021.128053

[13] Wang SY, Choi SH. Pareto-efficient coordination of the contract-based MTO supply chain under flexible cap-and-trade emission constraint. J Clean Prod. 2020;250:119571. doi: 10.1016/j.jclepro.2019.119571

[14] Ebrahimi S, Hosseini-Motlagh S-M, Nematollahi M, Cárdenas-Barrón LE. Coordinating double-level sustainability effort in a sustainable supply chain under cap-and-trade regulation. Expert Syst Appl. 2022;207:117872. doi: 10.1016/j.eswa.2022.117872

[15] Li J, Liang L, Xie J, Zhang G. Optimal emission reduction strategy for carbon neutral target: cap-and-trade policy and supply chain contracts with uncertain demand under SDG 13-climate action. Ann Oper Res. 2025. doi: 10.1007/s10479-025-06512-z

[16] Yan L, He P, Xu X, Chen J, Cheng TCEdwin. Platform operations with strategic inventory and green technology under the regional carbon cap-and-trade scheme. Ann Oper Res. 2025;359(2):1821–48. doi: 10.1007/s10479-024-06458-8

[17] Kang K, Tan BQ. Carbon emission reduction investment in sustainable supply chains under cap-and-trade regulation: an evolutionary game-theoretical perspective. Expert Syst with Appl. 2023;227:120335. doi: 10.1016/j.eswa.2023.120335

[18] Zhang X, Yousaf HMAU. Green supply chain coordination considering government intervention, green investment, and customer green preferences in the petroleum industry. J Clean Prod. 2020;246:118984. doi: 10.1016/j.jclepro.2019.118984

[19] He L, Wang H, Liu F. Emission abatement in low-carbon supply chains with government subsidy and information asymmetry. Int J Prod Res. 2024;62(18):6598–626. doi: 10.1080/00207543.2024.2305807

[20] Wu Z, Fan X, Zhu B, Xia J, Zhang L, Wang P. Do government subsidies improve innovation investment for new energy firms: A quasi-natural experiment of China’s listed companies. Technol Forecast Soc Change. 2022;175:121418. doi: 10.1016/j.techfore.2021.121418

[21] Quayson M, Chen W, Du H, Wang Z. A novel framework for evaluating the impact of carbon subsidies and taxes on Africa’s energy industry using optimization modelling. Environ Dev Sustain. 2024;27(11):26399–426. doi: 10.1007/s10668-024-04778-0

[22] Mridha B, Sarkar B. Implications of carbon policies for flexible demand and smart production with random lead time demand under a sustainable supply chain management. Environ Dev Sustain. 2025. doi: 10.1007/s10668-025-06038-1

[23] Sebatjane M. Inventory optimisation in a two-echelon cold chain: sustainable lot-sizing and shipment decisions under carbon cap emissions regulations. Ann Oper Res. 2025. doi: 10.1007/s10479-025-06466-2

[24] Zhao L, Yang C, Su B, Zeng S. Research on a single policy or policy mix in carbon emissions reduction. J Clean Prod. 2020;267:122030. doi: 10.1016/j.jclepro.2020.122030

[25] Zhou A, Li J. Impact of policy combinations on carbon emission performance: evidence from China. Clean Techn Environ Policy. 2024;26(9):3069–88. doi: 10.1007/s10098-024-02773-7

[26] Muthusamy P, Murugesan V, Selvaraj V. Optimal production–inventory decision with shortage for deterioration item and effect of carbon emission policy combination with green technology. Environ Dev Sustain. 2023;26(9):23701–66. doi: 10.1007/s10668-023-03621-2

[27] Choi SH, Wang XJ. Stochastic lot sizing for maximisation of shareholder wealth in make-to-order manufacturing. Int J Prod Res. 2014;53(4):1180–97. doi: 10.1080/00207543.2014.951090

[28] Hovelaque V, Bironneau L. The carbon-constrained EOQ model with carbon emission dependent demand. Int J Prod Econ. 2015;164:285–91. doi: 10.1016/j.ijpe.2014.11.022

[29] Gharaei A, Hoseini Shekarabi SA, Karimi M. Optimal lot-sizing of an integrated EPQ model with partial backorders and re-workable products: an outer approximation. Int J Syst Sci Oper Logist. 2021;10(1). doi: 10.1080/23302674.2021.2015007

[30] Sun L, Cao X, Alharthi M, Zhang J, Taghizadeh-Hesary F, Mohsin M. Carbon emission transfer strategies in supply chain with lag time of emission reduction technologies and low-carbon preference of consumers. J Clean Prod. 2020;264:121664. doi: 10.1016/j.jclepro.2020.121664

[31] Hong Z, Guo X. Green product supply chain contracts considering environmental responsibilities. Omega. 2019;83:155–66. doi: 10.1016/j.omega.2018.02.010

[32] Palczynsky B. Data centers – a threat to power supply or an opportunity for economic growth? The answer might be “yes.”. 2025. Available from: https://vestastrategicsolutions.com/blog/

[33] Graser S. BMW Group continues to drive electromobility: Long-term supply contract with Northvolt for battery cells from Europe concluded. 2020. Available from: https://www.press.bmwgroup.com/global/article/detail/T0311274EN/bmw-group-continues-to-drive-electromobility:-long-term-supply-contract-with-northvolt-for-battery-cells-from-europe-concluded?language=en

[34] Central University of Finance and Economics. Carbon allowance trading records of China in 2019. 2020. Available from: http://www.tanpaifang.com/tanzhibiao/202002/2968494.html

