# Supplementary material for: A game-theoretic analysis of production and coordination under combined carbon policies
Source: PLoS One. 2026 Apr 29;21(4):e0336358. doi: 10.1371/journal.pone.0336358 (PMC13127933; doi:10.1371/journal.pone.0336358)
Supplement: S2 File — (DOCX) [file pone.0336358.s002.docx]

**Appendix**

**Proof of Proposition 1.1**

**Step 1: Retailer’s Decision (Follower)**

The retailer aims to maximize profit *π_R_* by setting the retail price *p*. The profit function is given by:

$$\pi_{R}=(p-w-r)(a+gl-bp)H$$

The first-order and second-order derivatives with respect to *p* are:

$$\begin{matrix} \frac{\partial\pi_{R}}{\partial p}=-2bp+a+gl+b(w+r) \\ \frac{\partial^{2}\pi_{R}}{\partial p^{2}}=-2b \end{matrix}$$

Since *b*>0, we have $\frac{\partial^{2}\pi_{R}}{\partial p^{2}}<0$, ensuring *π_R_* is strictly concave in *p*. By setting $\partial\pi_{R}/\partial p=0$, we obtain the retailer’s optimal response function:

$$p\left(l\right)=\frac{a+gl+b\left(w+r\right)}{2b}$$

Substituting *p*(*l*) back into the demand function *D* = *a*+*gl*−*bp*, the effective demand

rate for the manufacturer becomes:

$$D\left(l\right)=\frac{a+gl-b\left(w+r\right)}{2}$$

**Step 2: Manufacturer’s Optimisation (Leader)**

The manufacturer maximizes profit *π_M_* by simultaneously determining the lot size *Q* and emission reduction amount *l*. Substituting *D*(*l*) and the lead time function *E*(*T*) into the manufacturer’s profit function, then we can calculate the partial derivatives.

*First-Order Conditions*: Differentiating *π_M_* with respect to *l* and *Q*, it follows:

$$\begin{matrix} \frac{\partial\pi_{M}}{\partial l}=\frac{gH}{2}(w-c-P_{E}e_{1}-\frac{S}{Q}-(h+P_{E}e_{2})(\frac{BQ}{2}+\frac{Q}{2\mu}+\frac{B}{2}+\tau+\frac{1}{2\mu}))-2(k-s)l+P_{E} \\ \frac{\partial\pi_{M}}{\partial Q}=\frac{H}{2}(\frac{S}{Q^{2}}-\frac{h+P_{E}e_{2}}{2}(B+\frac{1}{\mu}))(a+gl-b(w+r))-\frac{(h+P_{E}e_{2})H}{2} \end{matrix}$$

To analyse the optimal solution, the second-order conditions should be verified.

*Second-Order Conditions*: We derive the second partial derivatives to construct the Hessian Matrix *H*.

$$\begin{matrix} \frac{\partial^{2}\pi_{M}}{\partial l^{2}}=-2(k-s) \\ \frac{\partial^{2}\pi_{M}}{\partial Q^{2}}=-\frac{SH(a+gl-b(w+r))}{Q^{3}} \\ \frac{\partial^{2}\pi_{M}}{\partial Q\partial l}=\frac{\partial^{2}\pi_{M}}{\partial l\partial Q}=\frac{gH}{2}(\frac{S}{Q^{2}}-\frac{(h+P_{E}e_{2})}{2}(B+\frac{1}{\mu})) \end{matrix}$$

The Hessian Matrix is:

$$H=\left(\begin{matrix} \frac{\partial^{2}\pi_{M}}{\partial l^{2}} & \frac{\partial^{2}\pi_{M}}{\partial l\partial Q} \\ \frac{\partial^{2}\pi_{M}}{\partial Q\partial l} & \frac{\partial^{2}\pi_{M}}{\partial Q^{2}} \end{matrix}\right)=\left(\begin{matrix} -\frac{SH(a+gl-b(w+r))}{Q^{3}} & \frac{gH}{2}(\frac{S}{Q^{2}}-\frac{(h+P_{E}e_{2})}{2}(B+\frac{1}{\mu})) \\ \frac{gH}{2}(\frac{S}{Q^{2}}-\frac{(h+P_{E}e_{2})}{2}(B+\frac{1}{\mu})) & -2(k-s) \end{matrix}\right)$$

For *π_M_* to be jointly concave in (*Q*, *l*), the Hessian must be negative definite. This requires:

$$\begin{matrix} \frac{\partial^{2}\pi_{M}}{\partial Q^{2}}<0 \\ \frac{\partial^{2}\pi_{M}}{\partial l^{2}}<0 \\ \frac{\partial^{2}\pi_{M}}{\partial Q^{2}}\cdot\frac{\partial^{2}\pi_{M}}{\partial l^{2}}-\frac{\partial^{2}\pi_{M}}{\partial Q\partial l}\cdot\frac{\partial^{2}\pi_{M}}{\partial l\partial Q}>0 \end{matrix}$$

Condition 1 is satisfied since the demand is non-negative. Condition 2 is satisfied since the investment cost coefficient *k* is larger than the subsidy coefficient *s*. Condition 3 leads to the constraint presented in Proposition 1.1: 2*S*(*k-s*)(*a+gl-b*(*w+r*))*/Q^3^>g^2^H*(*S/Q^2^-*(*h+P_E_e_2_*)(*B+1/µ*)*/2*)*^2^*/4. Under this condition, *π_M_* is jointly concave in *Q* and *l*. The optimal lot size *Q_g_^*^* and emission reduction *l_g_^*^* are unique and can be found by setting Eq. (A.5) to zero.

**Step 3: Solution Derivation**

By setting $\partial\pi_{M}/\partial l=0$, we can express *l* in terms of *Q*:

$$l\left(Q\right)=\frac{gH}{4(k-s)}(w-c-P_{E}e_{1}-\frac{S}{Q}-(\frac{BQ}{2}+\frac{Q}{2\mu}+\frac{B}{2}+\tau+\frac{1}{2\mu})(h+P_{E}e_{2}))+\frac{P_{E}}{2(k-s)}$$

Substituting *l*(*Q*) into $\partial\pi_{M}/\partial Q=0$ yields the implicit function of *Q_g_^*^*, as shown in Eq. (6) in Proposition 1.1.

**Proof of Proposition 1.2**

**Step 1: Retailer’s Decision (Follower)**

The retailer aims to maximize profit *π_R_* by setting the retail price *p*. The profit function is given by:

$$\pi_{R}=(p-w-r)(a+gl-bp)H$$

The first-order and second-order derivatives with respect to *p* are:

$$\begin{matrix} \frac{\partial\pi_{R}}{\partial p}=-2bp+a+gl+b(w+r) \\ \frac{\partial^{2}\pi_{R}}{\partial p^{2}}=-2b \end{matrix}$$

Since *b*>0, we have $\frac{\partial^{2}\pi_{R}}{\partial p^{2}}<0$, ensuring *π_R_* is strictly concave in *p*. By setting $\partial\pi_{R}/\partial p=0$, we obtain the retailer’s optimal response function:

$$p\left(l\right)=\frac{a}{2b}+\frac{gl}{2b}+\frac{w+r}{2}$$

Substituting *p*(*l*) back into the demand function *D* = *a*+*gl*−*bp*, the effective demand

rate for the manufacturer becomes:

$$D\left(l\right)=\frac{a+gl-b\left(w+r\right)}{2}$$

**Step 2: Manufacturer’s Optimisation (Leader)**

The manufacturer maximizes profit *π_M_* by simultaneously determining the lot size *Q* and emission reduction amount *l*. Substituting *D*(*l*) and the lead time function *E*(*T*) into the manufacturer’s profit function, Eq. (9) is obtained. Then we can calculate the partial derivatives.

*First-Order Conditions*: Differentiating *π_M_* with respect to *l* and *Q*, it follows:

$$\begin{matrix} \frac{\partial\pi_{M}}{\partial l}=\frac{gH}{2}(w-c-P_{E}e_{1}+m-\frac{S}{Q}-(h+P_{E}e_{2})(\frac{BQ+B}{2}+\frac{Q+1}{2\mu}+\tau))-2kl+P_{E} \\ \frac{\partial\pi_{M}}{\partial Q}=\frac{H}{2}(\frac{S}{Q^{2}}-\frac{(h+P_{E}e_{2})}{2}(B+\frac{1}{\mu}))(a+gl-b(w+r))-\frac{(h+P_{E}e_{2})H}{2} \end{matrix}$$

To analyse the optimal solution, the second-order conditions should be verified.

*Second-Order Conditions*: We derive the second partial derivatives to construct the Hessian Matrix *H*.

$$\begin{matrix} \frac{\partial^{2}\pi_{M}}{\partial l^{2}}=-2k \\ \frac{\partial^{2}\pi_{M}}{\partial Q^{2}}=-\frac{SH(a+gl-b(w+r))}{Q^{3}} \\ \frac{\partial^{2}\pi_{M}}{\partial Q\partial l}=\frac{\partial^{2}\pi_{M}}{\partial l\partial Q}=\frac{gH}{2}(\frac{S}{Q^{2}}-\frac{(h+P_{E}e_{2})}{2}(B+\frac{1}{\mu})) \end{matrix}$$

The Hessian Matrix is:

$$H=\left(\begin{matrix} \frac{\partial^{2}\pi_{M}}{\partial l^{2}} & \frac{\partial^{2}\pi_{M}}{\partial l\partial Q} \\ \frac{\partial^{2}\pi_{M}}{\partial Q\partial l} & \frac{\partial^{2}\pi_{M}}{\partial Q^{2}} \end{matrix}\right)=\left(\begin{matrix} -\frac{SH(a+gl-b(w+r))}{Q^{3}} & \frac{gH}{2}(\frac{S}{Q^{2}}-\frac{(h+P_{E}e_{2})}{2}(B+\frac{1}{\mu})) \\ \frac{gH}{2}(\frac{S}{Q^{2}}-\frac{(h+P_{E}e_{2})}{2}(B+\frac{1}{\mu})) & -2k \end{matrix}\right)$$

For *π_M_* to be jointly concave in (*Q*, *l*), the Hessian must be negative definite. This requires:

$$\begin{matrix} \frac{\partial^{2}\pi_{M}}{\partial Q^{2}}<0 \\ \frac{\partial^{2}\pi_{M}}{\partial l^{2}}<0 \\ \frac{\partial^{2}\pi_{M}}{\partial Q^{2}}\cdot\frac{\partial^{2}\pi_{M}}{\partial l^{2}}-\frac{\partial^{2}\pi_{M}}{\partial Q\partial l}\cdot\frac{\partial^{2}\pi_{M}}{\partial l\partial Q}>0 \end{matrix}$$

Condition 1 is satisfied since the demand is non-negative. Condition 2 is satisfied since the investment cost coefficient *k* is larger than 0. Condition 3 leads to the constraint presented in Proposition 1.2: 2*kS*(*a+gl-b*(*w+r*))*/Q^3^>g^2^H*(*S/Q^2^-*(*h+P_E_e_2_*)(*B+1/µ*)*/*2)*2/*4. Under this condition, *π_M_* is jointly concave in *Q* and *l*. The optimal lot size *Q_p_^*^* and emission reduction *l_p_^*^* are unique and can be found by setting Eq. (A.14) to zero.

**Step 3: Solution Derivation**

By setting $\partial\pi_{M}/\partial l=0$, we can express *l* in terms of *Q*:

$$l=\frac{gH}{4k}(w-P_{E}e_{1}+m-c-\frac{S}{Q}-(h+P_{E}e_{2})(\frac{BQ}{2}+\frac{Q}{2\mu}+\frac{B}{2}+\tau+\frac{1}{2\mu}))+\frac{P_{E}}{2k}$$

Substituting *l*(*Q*) into $\partial\pi_{M}/\partial Q=0$ yields the implicit function of *Q_g_^*^*, as shown in Eq. (10) in Proposition 1.2.

**Proof of Proposition 2.1**

**Step 1: Retailer’s Decision (Follower)**

The profit function of the retailer is given by:

$$\pi_{R}=(p-w-r)(a+gl-bp)H-\lambda kl^{2}$$

The first-order and second-order derivatives with respect to *p* are:

$$\begin{matrix} \frac{\partial\pi_{R}}{\partial p}=-2bp+a+gl+b(w+r) \\ \frac{\partial^{2}\pi_{R}}{\partial p^{2}}=-2b \end{matrix}$$

Since *b*>0, we have $\frac{\partial^{2}\pi_{R}}{\partial p^{2}}<0$, ensuring *π_R_* is strictly concave in *p*. By setting $\partial\pi_{R}/\partial p=0$, we obtain the retailer’s optimal response function:

$$p\left(l\right)=\frac{a}{2b}+\frac{gl}{2b}+\frac{w+r}{2}$$

Substituting *p*(*l*) back into the demand function *D* = *a*+*gl*−*bp*, the effective demand

rate for the manufacturer becomes:

$$D\left(l\right)=\frac{a+gl-b\left(w+r\right)}{2}$$

**Step 2: Manufacturer’s Optimisation (Leader)**

The manufacturer maximizes profit *π_M_* by simultaneously determining the lot size *Q* and emission reduction amount *l*. Substituting *D*(*l*) and the lead time function *E*(*T*) into the manufacturer’s profit function, then we can calculate the partial derivatives.

*First-Order Conditions*: Differentiating *π_M_* with respect to *l* and *Q*, it follows:

$$\begin{matrix} \frac{\partial\pi_{M}}{\partial l}=\frac{gH}{2}(w-c-P_{E}e_{1}-\frac{S}{Q}-(h+P_{E}e_{2})(\frac{BQ+B}{2}+\frac{Q+1}{2\mu}+\tau))-2((1-\lambda)k-s)l+P_{E} \\ \frac{\partial\pi_{M}}{\partial Q}=\frac{H}{2}(\frac{S}{Q^{2}}-\frac{h+P_{E}e_{2}}{2}(B+\frac{1}{\mu}))(a+gl-b(w+r))-\frac{(h+P_{E}e_{2})H}{2} \end{matrix}$$

To analyse the optimal solution, the second-order conditions should be verified.

*Second-Order Conditions*: We derive the second partial derivatives to construct the Hessian Matrix *H*.

$$\begin{matrix} \frac{\partial^{2}\pi_{M}}{\partial l^{2}}=-2((1-\lambda)k-s) \\ \frac{\partial^{2}\pi_{M}}{\partial Q^{2}}=-\frac{SH(a+gl-b(w+r))}{Q^{3}} \\ \frac{\partial^{2}\pi_{M}}{\partial Q\partial l}=\frac{\partial^{2}\pi_{M}}{\partial l\partial Q}=\frac{gH}{2}(\frac{S}{Q^{2}}-\frac{(h+P_{E}e_{2})}{2}(B+\frac{1}{\mu})) \end{matrix}$$

The Hessian Matrix is:

$$H=\left(\begin{matrix} -\frac{SH(a+gl-b(w+r))}{Q^{3}} & \frac{gH}{2}(\frac{S}{Q^{2}}-\frac{h+P_{E}e_{2}}{2}(B+\frac{1}{\mu})) \\ \frac{gH}{2}(\frac{S}{Q^{2}}-\frac{h+P_{E}e_{2}}{2}(B+\frac{1}{\mu})) & -2((1-\lambda)k-s) \end{matrix}\right)$$

For *π_M_* to be jointly concave in (*Q*, *l*), the Hessian must be negative definite. This requires:

$$\begin{matrix} \frac{\partial^{2}\pi_{M}}{\partial Q^{2}}<0 \\ \frac{\partial^{2}\pi_{M}}{\partial l^{2}}<0 \\ \frac{\partial^{2}\pi_{M}}{\partial Q^{2}}\cdot\frac{\partial^{2}\pi_{M}}{\partial l^{2}}-\frac{\partial^{2}\pi_{M}}{\partial Q\partial l}\cdot\frac{\partial^{2}\pi_{M}}{\partial l\partial Q}>0 \end{matrix}$$

Condition 1 is satisfied since the demand is non-negative. Condition 2 is satisfied since the subsidy coefficient will not exceed the actual investment cost of the manufacturer. Condition 3 leads to the constraint presented in Proposition 2.1: 2((1*-λ*)*k-s*)*S*(*a+gl-b*(*w+r*))*/Q^3^>g^2^H*(*S/Q^2^-*(*h+P_E_e_2_*)(*B+*1*/µ*)*/*2)*2/*4. Under this condition, *π_M_* is jointly concave in *Q* and *l*. The optimal lot size *Q_1_^*^* and emission reduction *l_1_^*^* are unique and can be found by setting Eq. (A.23) to zero.

**Step 3: Solution Derivation**

By setting $\partial\pi_{M}/\partial l=0$, we can express *l* in terms of *Q*:

$$l=\frac{gH}{4(k-\lambda k-s)}(w-c-P_{E}e_{1}-\frac{S}{Q}-(\frac{BQ+B}{2}+\frac{Q+1}{2\mu}+\tau)(h+P_{E}e_{2}))+\frac{P_{E}}{2(k-\lambda k-s)}$$

Substituting *l*(*Q*) into $\partial\pi_{M}/\partial Q=0$ yields the implicit function of *Q_1_^*^*, as shown in Eq. (14) in Proposition 2.1.

**Proof of Proposition 2.2**

**Step 1: Retailer’s Decision (Follower)**

The profit function of the retailer is given by:

$$\pi_{R}=((1-\theta)p-w-r)(a+gl-bp)H$$

The first-order and second-order derivatives with respect to *p* are:

$$\begin{matrix} \frac{\partial\pi_{R}}{\partial p}=-2b(1-\theta)p+(1-\theta)(a+gl)+b(w+r) \\ \frac{\partial^{2}\pi_{R}}{\partial p^{2}}=-2b(1-\theta) \end{matrix}$$

Since *b*>0, 1-*θ*>0, we have $\frac{\partial^{2}\pi_{R}}{\partial p^{2}}<0$, ensuring *π_R_* is strictly concave in *p*. By setting $\partial\pi_{R}/\partial p=0$, we obtain the retailer’s optimal response function:

$$p\left(l\right)=\frac{a}{2b}+\frac{gl}{2b}+\frac{w+r}{2(1-\theta)}$$

Substituting *p*(*l*) back into the demand function *D* = *a*+*gl*−*bp*, the effective demand

rate for the manufacturer becomes:

$$D\left(l\right)=\frac{a+gl}{2}-\frac{b\left(w+r\right)}{2\left(1-\theta\right)}$$

**Step 2: Manufacturer’s Optimisation (Leader)**

The manufacturer maximizes profit *π_M_* by simultaneously determining the lot size *Q* and emission reduction amount *l*. Substituting *D*(*l*) and the lead time function *E*(*T*) into the manufacturer’s profit function, then we can calculate the partial derivatives.

*First-Order Conditions*: Differentiating *π_M_* with respect to *l* and *Q*, it follows:

$$\begin{matrix} \frac{\partial\pi_{M}}{\partial l}=(\frac{g^{2}\theta H}{2b}-2(k-s))l+P_{E}+\frac{g\theta H}{4b}(a-\frac{b(w+r)}{1-\theta})+\frac{gH}{2}(w-c-P_{E}e_{1}+\frac{\theta a}{2b}+\frac{\theta(w+r)}{2(1-\theta)}) \\ -\frac{gH}{2}(\frac{S}{Q}+(h+P_{E}e_{2})((\frac{B}{2}+\frac{1}{\mu})Q+\frac{B}{2}+\tau+\frac{1}{2\mu})) \\ \frac{\partial\pi_{M}}{\partial Q}=\frac{H}{2}(a+gl-\frac{b(w+r)}{1-\theta})(\frac{S}{Q^{2}}-\frac{(h+P_{E}e_{2})}{2}(B+\frac{1}{\mu}))-\frac{(h+P_{E}e_{2})H}{2} \end{matrix}$$

To analyse the optimal solution, the second-order conditions should be verified.

*Second-Order Conditions*: We derive the second partial derivatives to construct the Hessian Matrix *H*.

$$\begin{matrix} \frac{\partial^{2}\pi_{M}}{\partial l^{2}}=\frac{g^{2}\theta H}{2b}-2(k-s) \\ \frac{\partial^{2}\pi_{M2}}{\partial Q^{2}}=-\frac{SH}{Q^{3}}(a+gl-\frac{bw+br}{1-\theta}) \\ \frac{\partial\pi_{M}}{\partial Q\partial l}=\frac{\partial\pi_{M}}{\partial l\partial Q}=\frac{gH}{2}(\frac{S}{Q^{2}}-\frac{(h+P_{E}e_{2})}{2}(B+\frac{1}{\mu})) \end{matrix}$$

The Hessian Matrix is:

$$H=\left(\begin{matrix} -\frac{SH}{Q^{3}}(a+gl-\frac{bw+br}{1-\theta}) & \frac{gH}{2}(\frac{S}{Q^{2}}-\frac{(h+P_{E}e_{2})}{2}(B+\frac{1}{\mu})) \\ \frac{gH}{2}(\frac{S}{Q^{2}}-\frac{(h+P_{E}e_{2})}{2}(B+\frac{1}{\mu})) & \frac{g^{2}\theta H}{2b}-2(k-s) \end{matrix}\right)$$

For *π_M_* to be jointly concave in (*Q*, *l*), the Hessian must be negative definite. This requires:

$$\begin{matrix} \frac{\partial^{2}\pi_{M}}{\partial Q^{2}}<0 \\ \frac{\partial^{2}\pi_{M}}{\partial l^{2}}<0 \\ \frac{\partial^{2}\pi_{M}}{\partial Q^{2}}\cdot\frac{\partial^{2}\pi_{M}}{\partial l^{2}}-\frac{\partial^{2}\pi_{M}}{\partial Q\partial l}\cdot\frac{\partial^{2}\pi_{M}}{\partial l\partial Q}>0 \end{matrix}$$

Condition 1 is satisfied since the demand is non-negative. Condition 2 and Condition 3 leads to the constraint presented in Proposition 2.2:

2*k-*2*s-θg^2^H/*2*b*>0 and (2*k-*2*s-θg^2^H/*2*b*)*S*(*a+gl-b*(*w+r*)/(1-*θ*))*/Q^3^>g^2^H*(*S/Q^2^-*(*h+P_E_e_2_*)(*B+*1*/µ*)*/*2)*2/*4.

Under this condition, *π_M_* is jointly concave in *Q* and *l*. The optimal lot size *Q_p_^*^* and emission reduction *l_p_^*^* are unique and can be found by setting Eq. (A.31) to zero.

**Step 3: Solution Derivation**

By setting $\partial\pi_{M}/\partial l=0$, we can express *l* in terms of *Q*:

$$\begin{matrix} l=\frac{2b}{4b(k-s)-\theta g^{2}H}(P_{E}+\frac{gHcon_{1}}{2}+\frac{g\theta H}{4b}(a-\frac{b(w+r)}{1-\theta})) \\ -\frac{bgH}{4b(k-s)-\theta g^{2}H}(\frac{S}{Q}+(h+P_{E}e_{2})(\frac{Q}{2}(B+\frac{1}{\mu})+\frac{B}{2}+\tau+\frac{1}{2\mu})) \\ where \\ con_{1}=w-c-P_{E}e_{1}+\frac{\theta a}{2b}+\frac{\theta(w+r)}{2(1-\theta)} \end{matrix}$$

Substituting *l*(*Q*) into $\partial\pi_{M}/\partial Q=0$ yields the implicit function of *Q_2_^*^*, as shown in Eq. (18) in Proposition 2.2.

**Proof of Proposition 2.3**

**Step 1: Retailer’s Decision (Follower)**

The profit function of the retailer is given by:

$$\pi_{R}=(p-w-r)(a+gl-bp)H-\lambda kl^{2}$$

The first-order and second-order derivatives with respect to *p* are:

$$\begin{matrix} \frac{\partial\pi_{R}}{\partial p}=-2bp+a+gl+b(w+r) \\ \frac{\partial^{2}\pi_{R}}{\partial p^{2}}=-2b \end{matrix}$$

Since *b*>0, we have $\frac{\partial^{2}\pi_{R}}{\partial p^{2}}<0$, ensuring *π_R_* is strictly concave in *p*. By setting $\partial\pi_{R}/\partial p=0$, we obtain the retailer’s optimal response function:

$$p\left(l\right)=\frac{a}{2b}+\frac{gl}{2b}+\frac{w+r}{2}$$

Substituting *p*(*l*) back into the demand function *D* = *a*+*gl*−*bp*, the effective demand

rate for the manufacturer becomes:

$$D\left(l\right)=\frac{a+gl-b\left(w+r\right)}{2}$$

**Step 2: Manufacturer’s Optimisation (Leader)**

The manufacturer maximizes profit *π_M_* by simultaneously determining the lot size *Q* and emission reduction amount *l*. Substituting *D*(*l*) and the lead time function *E*(*T*) into the manufacturer’s profit function, then we can calculate the partial derivatives.

*First-Order Conditions*: Differentiating *π_M_* with respect to *l* and *Q*, it follows:

$$\begin{matrix} \frac{\partial\pi_{M}}{\partial l}=\frac{gH}{2}(w-c+m-P_{E}e_{1}-\frac{S}{Q}-(h+P_{E}e_{2})(\frac{BQ+B}{2}+\frac{Q+1}{2\mu}+\tau))-2(1-\lambda)kl+P_{E} \\ \frac{\partial\pi_{M}}{\partial Q}=\frac{H}{2}(\frac{S}{Q^{2}}-\frac{h+P_{E}e_{2}}{2}(B+\frac{1}{\mu}))(a+gl-b(w+r))-\frac{(h+P_{E}e_{2})H}{2} \end{matrix}$$

To analyse the optimal solution, the second-order conditions should be verified.

*Second-Order Conditions*: We derive the second partial derivatives to construct the Hessian Matrix *H*.

$$\begin{matrix} \frac{\partial^{2}\pi_{M}}{\partial l^{2}}=-2(1-\lambda)k \\ \frac{\partial^{2}\pi_{M}}{\partial Q^{2}}=-\frac{SH(a+gl-b(w+r))}{Q^{3}} \\ \frac{\partial^{2}\pi_{M}}{\partial Q\partial l}=\frac{\partial^{2}\pi_{M}}{\partial l\partial Q}=\frac{gH}{2}(\frac{S}{Q^{2}}-\frac{(h+P_{E}e_{2})}{2}(B+\frac{1}{\mu})) \end{matrix}$$

The Hessian Matrix is:

$$H=\left(\begin{matrix} -\frac{SH(a+gl-b(w+r))}{Q^{3}} & \frac{gH}{2}(\frac{S}{Q^{2}}-\frac{h+P_{E}e_{2}}{2}(B+\frac{1}{\mu})) \\ \frac{gH}{2}(\frac{S}{Q^{2}}-\frac{h+P_{E}e_{2}}{2}(B+\frac{1}{\mu})) & -2(1-\lambda)k \end{matrix}\right)$$

For *π_M_* to be jointly concave in (*Q*, *l*), the Hessian must be negative definite. This requires:

$$\begin{matrix} \frac{\partial^{2}\pi_{M}}{\partial Q^{2}}<0 \\ \frac{\partial^{2}\pi_{M}}{\partial l^{2}}<0 \\ \frac{\partial^{2}\pi_{M}}{\partial Q^{2}}\cdot\frac{\partial^{2}\pi_{M}}{\partial l^{2}}-\frac{\partial^{2}\pi_{M}}{\partial Q\partial l}\cdot\frac{\partial^{2}\pi_{M}}{\partial l\partial Q}>0 \end{matrix}$$

Condition 1 is satisfied since demand is non-negative. Condition 2 is satisfied since *λ*<1. Condition 3 leads to the constraint presented in Proposition 2.3: 2*S*(1*-λ*)*k*(*a+gl-b*(*w+r*))*/Q^3^>g^2^H*(*S/Q^2^-*(*h+P_E_e_2_*)(*B+*1*/µ*)*/*2)*2/*4. Under this condition, *π_M_* is jointly concave in *Q* and *l*. The optimal lot size *Q_3_^*^* and emission reduction *l_3_^*^* are unique and can be found by setting Eq. (A.41) to zero.

**Step 3: Solution Derivation**

By setting $\partial\pi_{M}/\partial l=0$, we can express *l* in terms of *Q*:

$$l=\frac{gH}{4(1-\lambda)k}(w-c+m-P_{E}e_{1}-\frac{S}{Q}-(\frac{BQ+B}{2}+\frac{Q+1}{2\mu}+\tau)(h+P_{E}e_{2}))+\frac{P_{E}}{2(1-\lambda)k}$$

Substituting *l*(*Q*) into $\partial\pi_{M}/\partial Q=0$ yields the implicit function of *Q_3_^*^*, as shown in Eq. (22) in Proposition 2.3.

**Proof of Proposition 2.4**

**Step 1: Retailer’s Decision (Follower)**

The profit function of the retailer is given by:

$$\pi_{R}=((1-\theta)p-w-r)(a+gl-bp)H$$

The first-order and second-order derivatives with respect to *p* are:

$$\begin{matrix} \frac{\partial\pi_{R}}{\partial p}=-2b(1-\theta)p+(1-\theta)(a+gl)+b(w+r) \\ \frac{\partial^{2}\pi_{R}}{\partial p^{2}}=-2b(1-\theta) \end{matrix}$$

Since *b*>0, 1-*θ*>0, we have $\frac{\partial^{2}\pi_{R}}{\partial p^{2}}<0$, ensuring *π_R_* is strictly concave in *p*. By setting $\partial\pi_{R}/\partial p=0$, we obtain the retailer’s optimal response function:

$$p\left(l\right)=\frac{a}{2b}+\frac{gl}{2b}+\frac{w+r}{2(1-\theta)}$$

Substituting *p*(*l*) back into the demand function *D* = *a*+*gl*−*bp*, the effective demand

rate for the manufacturer becomes:

$$D\left(l\right)=\frac{a+gl}{2}-\frac{b\left(w+r\right)}{2\left(1-\theta\right)}$$

**Step 2: Manufacturer’s Optimisation (Leader)**

The manufacturer maximizes profit *π_M_* by simultaneously determining the lot size *Q* and emission reduction amount *l*. Substituting *D*(*l*) and the lead time function *E*(*T*) into the manufacturer’s profit function, then we can calculate the partial derivatives.

*First-Order Conditions*: Differentiating *π_M_* with respect to *l* and *Q*, it follows:

$$\begin{matrix} \frac{\partial\pi_{M}}{\partial l}=(\frac{g^{2}\theta H}{2b}-2k)l+P_{E}+\frac{g\theta H}{4b}(a-\frac{b(w+r)}{1-\theta})+\frac{gH}{2}(w-c+m-P_{E}e_{1}+\frac{\theta a}{2b}+\frac{\theta(w+r)}{2(1-\theta)}) \\ -\frac{gH}{2}(\frac{S}{Q}+(h+P_{E}e_{2})((\frac{B}{2}+\frac{1}{\mu})Q+\frac{B}{2}+\tau+\frac{1}{2\mu})) \\ \frac{\partial\pi_{M}}{\partial Q}=\frac{H}{2}(a+gl-\frac{b(w+r)}{1-\theta})(\frac{S}{Q^{2}}-\frac{(h+P_{E}e_{2})}{2}(B+\frac{1}{\mu}))-\frac{(h+P_{E}e_{2})H}{2} \end{matrix}$$

To analyse the optimal solution, the second-order conditions should be verified.

*Second-Order Conditions*: We derive the second partial derivatives to construct the Hessian Matrix *H*.

$$\begin{matrix} \frac{\partial^{2}\pi_{M}}{\partial l^{2}}=\frac{g^{2}\theta H}{2b}-2k \\ \frac{\partial^{2}\pi_{M2}}{\partial Q^{2}}=-\frac{SH}{Q^{3}}(a+gl-\frac{bw+br}{1-\theta}) \\ \frac{\partial\pi_{M}}{\partial Q\partial l}=\frac{\partial\pi_{M}}{\partial l\partial Q}=\frac{gH}{2}(\frac{S}{Q^{2}}-\frac{h+P_{E}e_{2}}{2}(B+\frac{1}{\mu})) \end{matrix}$$

The Hessian Matrix is:

$$H=\left(\begin{matrix} -\frac{SH}{Q^{3}}(a+gl-\frac{bw+br}{1-\theta}) & \frac{gH}{2}(\frac{S}{Q^{2}}-\frac{h+P_{E}e_{2}}{2}(B+\frac{1}{\mu})) \\ \frac{gH}{2}(\frac{S}{Q^{2}}-\frac{h+P_{E}e_{2}}{2}(B+\frac{1}{\mu})) & \frac{g^{2}\theta H}{2b}-2k \end{matrix}\right)$$

For *π_M_* to be jointly concave in (*Q*, *l*), the Hessian must be negative definite. This requires:

$$\begin{matrix} \frac{\partial^{2}\pi_{M}}{\partial Q^{2}}<0 \\ \frac{\partial^{2}\pi_{M}}{\partial l^{2}}<0 \\ \frac{\partial^{2}\pi_{M}}{\partial Q^{2}}\cdot\frac{\partial^{2}\pi_{M}}{\partial l^{2}}-\frac{\partial^{2}\pi_{M}}{\partial Q\partial l}\cdot\frac{\partial^{2}\pi_{M}}{\partial l\partial Q}>0 \end{matrix}$$

Condition 1 is satisfied since the demand is non-negative. Condition 2 and Condition 3 leads to the constraint presented in Proposition 2.4:

*S*(2*k-θg^2^H/*2*b*)(*a+gl-b*(*w+r*)*/*(*1-θ*))*/Q^3^>g^2^H*(*S/Q^2^-*(*h+P_E_e_2_*)(*B+1/µ*)*/*2)*^2^*/4 and *k-θg^2^H/b>*0.

Under this condition, *π_M_* is jointly concave in *Q* and *l*. The optimal lot size *Q_4_^*^* and emission reduction *l_4_^*^* are unique and can be found by setting Eq. (A.50) to zero.

**Step 3: Solution Derivation**

By setting $\partial\pi_{M}/\partial l=0$, we can express *l* in terms of *Q*:

$$\begin{matrix} l=\frac{2b}{4bk-\theta g^{2}H}(P_{E}+\frac{gHcon_{2}}{2}+\frac{g\theta H}{4b}(a-\frac{b(w+r)}{1-\theta})) \\ -\frac{bgH}{4bk-\theta g^{2}H}(\frac{S}{Q}+(h+P_{E}e_{2})(\frac{Q}{2}(B+\frac{1}{\mu})+\frac{B}{2}+\tau+\frac{1}{2\mu})) \\ where \\ con_{2}=w-c+m-P_{E}e_{1}+\frac{\theta a}{2b}+\frac{\theta(w+r)}{2(1-\theta)} \end{matrix}$$

Substituting *l*(*Q*) into $\partial\pi_{M}/\partial Q=0$ yields the implicit function of *Q_4_^*^*, as shown in Eq. (25) in Proposition 2.4.
