# Supplementary figures and images for: A game-theoretic analysis of production and coordination under combined carbon policies
Source: PLoS One. 2026 Apr 29;21(4):e0336358. doi: 10.1371/journal.pone.0336358 (PMC13127933; doi:10.1371/journal.pone.0336358)

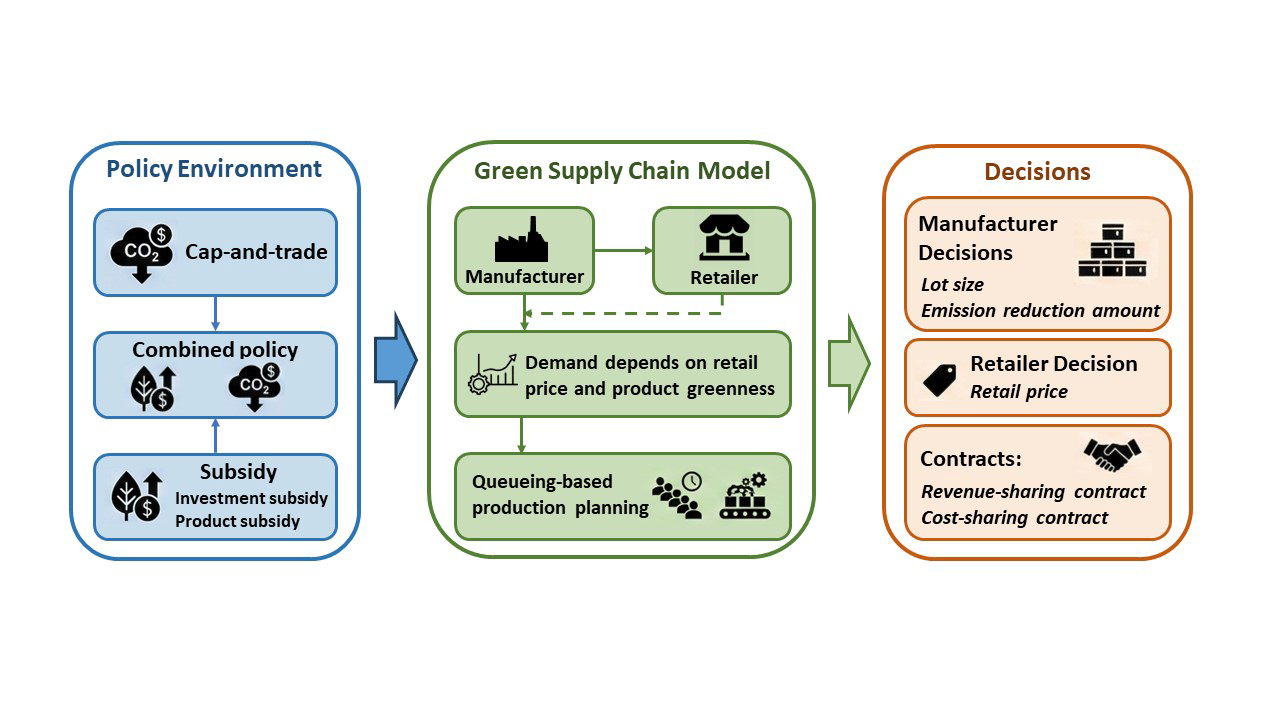

Supplement: S1 Fig — (TIFF) [file pone.0336358.s003.tiff]
